# Automating life science labs at the single-cell level through precise ultrasonic liquid sample ejection: PULSE

**DOI:** 10.1038/s41378-024-00798-y

**Published:** 2024-11-20

**Authors:** Peiran Zhang, Zhenhua Tian, Ke Jin, Kaichun Yang, Wesley Collyer, Joseph Rufo, Neil Upreti, Xianjun Dong, Luke P. Lee, Tony Jun Huang

**Affiliations:** 1 Department of Mechanical Engineering and Materials Science, Duke University, Durham, NC 27708 USA; 2 Department of Mechanical Engineering, Virginia Polytechnic Institute and State University, Blacksburg, VA 24061 USA; 3 Genomics and Bioinformatics Hub, Department of Neurology, Brigham and Women’s Hospital, Harvard Medical School, Boston, MA USA; 4 Department of Medicine, Brigham and Women’s Hospital, Harvard Medical School, Boston, MA USA; 5 Department of Bioengineering, Department of Electrical Engineering and Computer Science, University of California at Berkeley, Berkeley, CA USA; 6 Institute of Quantum Biophysics, Department of Biophysics, Sungkyunkwan University, Suwon, Korea; 7 Department of Chemistry & Nanoscience, Ewha Womans University, Seoul, Korea

**Keywords:** Microfluidics, Engineering

## Abstract

Laboratory automation technologies have revolutionized biomedical research. However, the availability of automation solutions at the single-cell level remains scarce, primarily owing to the inherent challenges of handling cells with such small dimensions in a precise, biocompatible manner. Here, we present a single-cell-level laboratory automation solution that configures various experiments onto standardized, microscale test-tube matrices via our precise ultrasonic liquid sample ejection technology, known as PULSE. PULSE enables the transformation of titer plates into microdroplet arrays by printing nanodrops and single cells acoustically in a programmable, scalable, and biocompatible manner. Unlike pipetting robots, PULSE enables researchers to conduct biological experiments using single cells as anchoring points (e.g., 1 cell *vs*. 1000 cells per “tube”), achieving higher resolution and potentially more relevant data for modeling and downstream analyses. We demonstrate the ability of PULSE to perform biofabrication, precision gating, and deterministic array barcoding via preallocated droplet-addressable primers. Single cells can be gently printed at a speed range of 5–20 cell⋅s^−1^ with an accuracy of 90.5–97.7%, which can then adhere to the substrate and grow for up to 72 h while preserving cell integrity. In the deterministic barcoding experiment, 95.6% barcoding accuracy and 2.7% barcode hopping were observed by comparing the phenotypic data with known genotypic data from two types of single cells. Our PULSE platform allows for precise and dynamic analyses by automating experiments at the single-cell level, offering researchers a powerful tool in biomedical research.

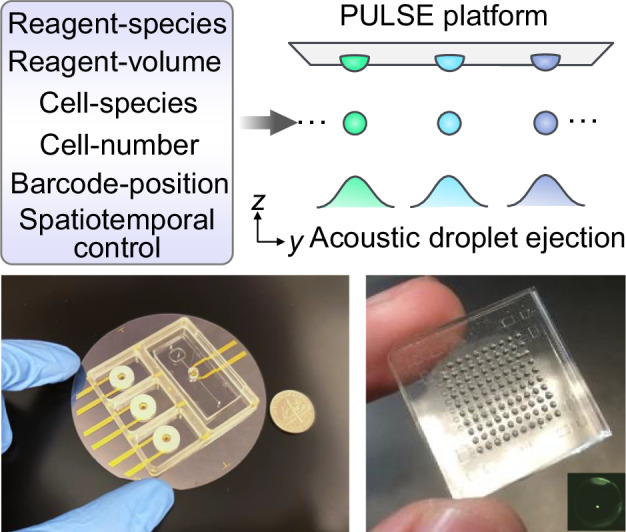

## Introduction

Lab automation promotes advancements in biomedical research^1–5^ while also being a crucial driver for improving the precision and reproducibility of experiments. Recent advances in liquid handling techniques, including LabDroids^6,7^, self-driving laboratories^8–12^, centralized cloud labs^13–15^, and lab-on-a-chip platforms^16–46^, have demonstrated a clear trend toward the automation of data collection due to their unrivaled speed, consistency, and level of integration. However, since most current automation solutions focus on bulk-cell samples, the associated limitations, including the population mask, undesired cooperative behavior, and ambiguity of sample compositions, add significant uncertainties within the collected data.

In contrast, a single-cell, as a complete carrier of multi-omic information^47,48^, is easily interpretable by models^48,49^ and can serve as a good anchor point for automating data collection in various scenarios^1,50^, including rare-event discovery, monoclonal-related production, directed evolution, and artificial intelligence (AI)-guided learning. Over the past decade, several single-cell tools^51^ have been developed via this methodology, including 10x Genomics, spatial-omics, CellenONE, Celldom, Cytena, Namocell, CellShepherd, and DispenCell. Despite their successes, the degree of automation, integration potential, and flexibility have not reached the standards of existing automation technologies, and the power of single-cell-level automation has not been fully realized in biomedical research. There lies a vast space of unaddressed opportunities for developing dexterous and integrated solutions for lab automation at the single-cell level.

**Fig. 1:**
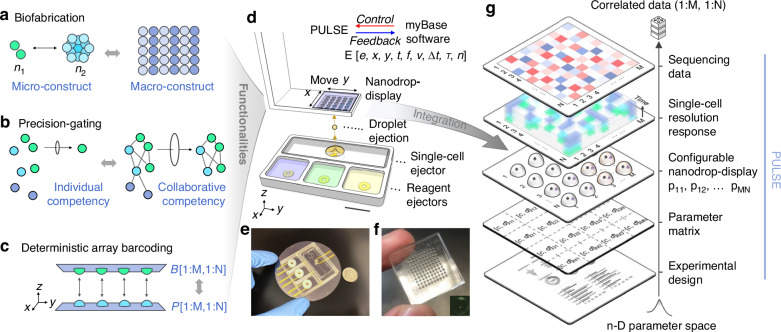
PULSE: an acoustic-based automated single-cell-analysis platform. **a** Biofabrication *via* PULSE from the microscale to the macroscale. *n*, particle number. **b** Precision gating *via* PULSE for single-cells and bulk-cells. **c** Deterministic array barcoding of known cells via known primers. **d** Schematic overview of the dispensing module of the PULSE platform. “myBase”: homemade software for controlling PULSE over various parameters in vector **E**. Scale bar: 1 cm. **e** A PULSE cartridge integrating an acoustic-based multicolor single-cell ejector and three independent reagent ejectors on a piezoelectric substrate. A circularly confined ring disc is placed over the ejector to constrain the liquid meniscus and dampen the water waves. **f** Aligned printing of nanodrops into microwells. Inset: an ejected droplet containing one fluorescent particle. Scale bar: 200 µm. **g** PULSE deterministically threads the whole pipeline of quantitative precision biological experiments with single-cell resolution, from experimental design, parameter matrix formation, and nanodrop printing to acquiring single-cell response and sequencing data on a compact device. [*c*, *d*]: *c*, the vector of cell types and numbers; *d*, the vector of drug types and concentrations. “n-D”, n-dimensional

Here, we present precise ultrasonic liquid sample ejection: PULSE (Fig. 1), an integrated lab automation solution configuring different experiments to standardized, microscale test-tube matrices via our acoustic ejection technique. With PULSE, we deterministically print various reagents and single cells into nanodrop-pixel arrays, creating an automated and scalable process. We employ self-focusing acoustic waves to eject droplets (0.2 pL to 10 μL per drop, 200 to 40,000 droplets per second with the volume and the throughput dependent on ejector designs, excitation signals, and liquid properties) through the use of interdigital transducers made by standard microfabrication techniques, allowing more compact ejectors, greater scalability, a larger volumetric range, and disposable ejectors compared with state-of-the-art bulk-acoustic-wave liquid handlers (e.g., ECHO).

Owing to the gentle nature of ultrasound and well-controlled microenvironments, PULSE demonstrates high biocompatibility even among state-of-the-art automation technologies (e.g., 0–8 genes are significantly regulated 1–24 h postejection), maximizing the likelihood of maintaining the native states of biological samples. In this work, we demonstrate the capability of PULSE from three aspects. First, the fabrication of clusters, spheroids, and hydrogel patterns can be automatically carried out via different printing logics with excellent reconfigurability (Fig. 1a). Second, the precision control, observation, and selection (i.e., gating) of individual cells (Fig. 1b) are accomplished via PULSE on individual nanodrops, allowing researchers to investigate the heterogeneity and rare events among population cells. Third, the deterministic array barcoding of single cells (Fig. 1c) overrides Poisson’s distribution and directly bridges the gap between phenotypes and genotypes with preallocated droplet-addressable primers. From an application perspective, an important unmet need for automated single-cell assays is the testing and observation of target single cells within multiplexed, controlled, and orthogonal conditions in a deterministic manner, preferably with direct interfaces to single-cell sequencing. PULSE opens up the possibility of fully integrating individual cells, test conditions, data collection, sample retrieval, and sequencing via dynamically configurable open microreactor arrays, thus allowing researchers to perform orthogonal and deterministic single-cell assays such as current automation technologies that use bulk-cell samples.

PULSE can handle the major stages of biological experimentations, from experimental design to the acquisition of phenotypes and genotypes, in a deterministic manner. It can potentially facilitate single-cell-level studies in the life sciences, including experiments in the fields of embryogenesis^52^, assembloids^53^, tissue engineering^54^, immunology^55^, synthetic biology^56,57^, evolutionary dynamics^58,59^, and self-driving labs^12^, with precision, scalability, and flexibility.

## Results

### PULSE: automation, integration, and standardization at the single-cell level

The principal working mechanism of PULSE (Fig. 1) is scaling down titer plate-based experiments into microscale test matrices by printing nanodrops with single-cell-level resolution and single droplet configurability (Fig. 1d). The PULSE cartridge, as shown in Fig. 1e, Figs. S2 and S3, consists of one multicolor single-cell dispenser and three reagent dispensers, each equipped with three open reservoirs (Fig. 1e). These acoustic dispensers are compact, nozzle-free, biocompatible, and easily scaled up (by one-layer lithography). Using this cartridge, different types of single cells and reagents are programmatically deposited into a nanodrop-pixel array on the receiver substrates (“nanodrop-display”, Fig. 1f) with precise control of the particle number and droplet volume. This feature enables diverse functionalities, including biofabrication, cloning, precision gating, and array barcoding, with pixel-level reconfigurability. Furthermore, the design strategy of PULSE is to thread the major stages of bioexperiments in an automation-compatible manner (Fig. 1g), including test-matrix translation, nanodrop deposition, cell cultivation, measurements, sequencing, and product retrieval. Following this strategy, one can retrace their steps at the single-cell level and correlate the data of each droplet using its location in the array.

**Fig. 2:**
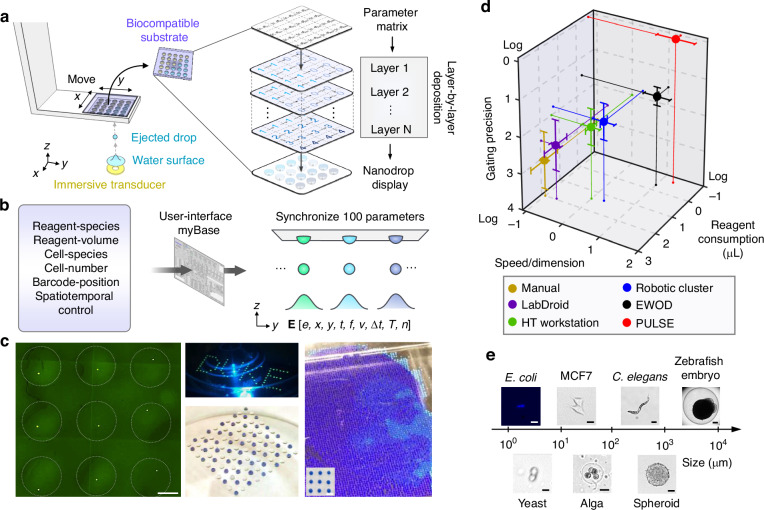
PULSE transforms the experimental test design into a nanodrop-pixel array *via* dynamic droplet deposition. **a** Schematic process outlining the transformation of the parameter matrix to the execution steps for nanodrop display deposition. The numbers in the layers represent the number of repetitions of ejection. **b** “myBase” software interfaces with the user and synchronizes >100 parameters in experiments. **E** [*e*, *x*, *y*, *t*, *f*, *v*, Δ*t*, *τ*, *n*]: **E**, parameter vector; *e*, ejector index; *x* and *y*, the 2D coordinates of the motorized stage; *t*, the timing of dispensing; *f*, *v*, Δ*t*, *τ*, and *n*, the frequency, amplitude, delay, duration, and repetition of the acoustic pulses. **c** Examples of nanodrop displays with different printing configurations. Scale bars: 400 µm. **d** Three-dimensional comparison of the gating precision, speed, characteristic dimension, and reagent consumption of PULSE with other methods. HT Workstation, standalone high-throughput workstation. Robotic Cluster, integrated automation infrastructure for bio-manufacturing similar to BioFoundry. EWOD, digital microfluidics based on electrowetting-on-dielectrics. **e** Various biosamples ejected by PULSE, ranging from bacteria (~1 μm) to model organisms (~1 mm). Scale bars: *E*. *coli* – MCF7: 5 µm; Alga – Zebrafish Embryo: 50 µm

Once an experimental design is imported, it is translated into a standard test matrix containing the collective information of the execution details, including the ejector index (*e*), stage position (*x*, *y*), pulse repetition number (*n*), and pulse duration (*τ*). This test matrix is further compiled into multiple layers of execution code. Each layer of the code coordinates the dispensing of one specific type of reagent/cell, which is finally printed into the nanodrop display under the desired experimental conditions (Fig. 2a). The homemade software “myBase” (Fig. 2b, Supplementary Note S1, and Supplementary Fig. S1), which interfaces with the biologists in terms of the experimental details and synchronizes over 100 parameters during deposition, was developed to coordinate the whole process. Using myBase, the nanodrop display can be dynamically configured to adapt to a wide variety of experimental designs. Figure 2c shows the versatility of PULSE, which illustrates the custom nanodrop-display patterns, including a single-cell array, “DUKE” letters, a checkerboard pattern, and a six-color image. We evaluated the performance of PULSE compared with other major automated solutions, including LabDroids, high-throughput workstations (e.g., stamping liquid handlers), centralized laboratories (e.g., cloud labs with integrated liquid-handling robots), and microfluidics (e.g., electrowetting-based devices)^60^ (Fig. 2d). From the perspective of Fig. 2d, PULSE has the advantages over these automated solutions in terms of gating precision (single-cell level), speed (20–200 event⋅s^−1^), and reagent consumption (~200 nL per pixel for the cell assay).

Notably, compared with standalone single-cell analysis instruments such as FACS or other single-cell dispensers, PULSE has the same level of gating precision (i.e., 1 cell) in terms of the dispensing resolution, downstream observation resolution, and sample collection resolution. Furthermore, owing to its nozzle-free design, PULSE can eject biological samples of various shapes or sizes (Fig. 2e), including *E*. *coli* (~1 μm), *S. cerevisiae* (~5 μm), MCF7 cells (~10 μm), algae (~30 μm), spheroids (~200 μm), *D. rerio* embryos (~1 mm), and *C. elegans* (~2 mm in length).

### Acoustic dispensing *via* PULSE with single-cell resolution

**Fig. 3:**
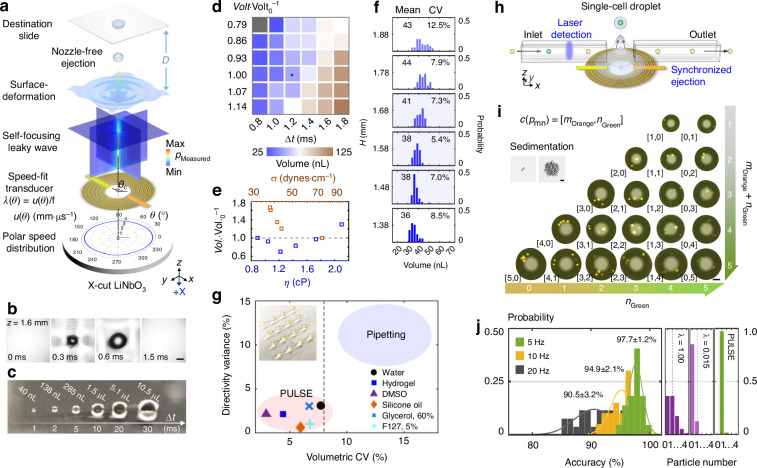
Flexible dispensing of reagents and single cells *via* PULSE. **a** Design principle of the speed-fit interdigital transducer for high-precision acoustic dispensing. The three-dimensional cross-sectional view of the acoustic pressure is measured by a hydrophone. *θ*, rotation angle with respect to the -*X* face of LiNbO_3_. *u*, wave speed. *θ*_*c*_, rotation angle for the electrode connectors; 135° for the -*X* face of LiNbO_3_. *D*, distance, 7–50 mm, typically 9 mm. **b** Time-lapse images of water-surface deformation upon ejection. Scale bar: 80 µm. **c** The board dynamic range of the nanodrop volume in one ejection event by the same ejector, from 40 nL to 10,500 nL. **d** The volume of the nanodrop-pixel is continuously tunable by the excitation amplitude (*Volt*.⋅Volt._0_^−1^) and duration (Δ*t*). The asterisk (*) indicates the optimal condition in terms of directivity and volumetric consistency. **e** Impact of viscosity (*η*) and surface tension (*σ*) on the droplet volume (*Vol*.⋅Vol._0_^−1^). **f** Volume distribution with respect to the water height. Blue shading: typical water height for the ejector. **g** Printing different types of fluid *via* PULSE. Inset: ejected silicone oil array containing two fluorescent aqueous droplets that undergo sedimentation and are later merged. **h** Schematic mechanism of deterministic single-cell ejection *via* PULSE. **i** Precise control of the number of particles in individual nanodrop-pixels *via* PULSE. Insets: particle pairs and clusters in inverted nanodrop pixels due to sedimentation. Scale bars: left, 30 µm; right, 100 µm. **j** Validation of single-cell accuracy at dispensing speeds of 5, 10, and 20 single-cell-droplet per second. Right, the comparison between stochastic Poisson distributions (purple) and PULSE (green) on the occupancy percentage in the nanodrops

PULSE employs speed-fitting interdigital transducers (Fig. 3a) to achieve high-throughput, high-precision, and highly tunable acoustic dispensing. The transducer consists of annular-comb-shaped, alternating metallic fingers deposited on a piezoelectric substrate (i.e., *X*-cut LiNbO_3_) immersed in fluid. Once a sinusoidal electrical pulse is applied to those alternating fingers, surface acoustic waves are generated and leak into the fluid (leaky waves) in a direction following Rayleigh’s law^61^ (i.e., sin^−1^(c_water_⋅c_substrate_(*θ*)^−1^)) (Supplementary Movie S1). Owing to the fine-tuned annular arrangement of the transducer fingers, those leaky waves converge at the designated focal point, impinge on the fluid‒air interface, and gently eject a small volume of fluid out of the interface upward upon highly localized pressure (Fig. 3b, Supplementary Fig. S2, and Movie S2). The design consists of alternating antisymmetric metallic fingers (Supplementary Note S2) with an arrangement that matches the anisotropic speed distribution and the related phase delays of the piezoelectric substrate (Fig. 3a). Therefore, phase synchronization and maximal acoustic amplitudes are obtained at the focal point for efficient ejection. Using a speed-fitting transducer operating at 20.4 MHz, an extensive dynamic range of droplet volume is allowed with different pulse durations, ranging from 40 nL to 10.5 μL, in a single-ejection event (Fig. 3c), and a maximal throughput of 200 event⋅s^−1^ is achieved.

To improve robustness, we optimized key performance indicators, such as the volumetric variance, directivity, tunability, and throughput, to ensure high consistency and precision. To this end, an array of parameters, including excitation amplitudes, pulse durations (Fig. 3d), fluid surface tensions, viscosities (Fig. 3e), fluid layer thicknesses (Fig. 3f), and reagent meniscuses, were also optimized to achieve optimal acoustic dispensing performance. As a result, liquids with different physical properties, including surface tension, viscosity, acoustic attenuation, and electrical conductivity, can be dispensed with high consistency (coefficient of variation: 2–7%) and directivity (variance: 2–3%) relative to gold-standard pipetting methods (Fig. 3g), especially within the submicroliter dispensing scenarios. For example, aqueous nanodrops can be sequentially ejected into a preprinted mineral oil droplet array for merging without solid‒liquid contact (Fig. 3g, inset). Among existing liquid dispensers, our liquid ejector is compact (planar, <3 mm), scalable (fabricated by traditional one-mask-layer lithography), energy efficient (optimized piezoelectric material), durable, and allows ultralow volume dispensing. In certain scenarios, by optimizing the working frequency (e.g., 180 MHz) and geometry of the ejector, the minimal droplet volume and the dead volume have been reduced to 0.2 pL and 0.3 μL, respectively, at 40,000 droplets⋅s^−1^, indicating its promising potential for dispensing small-volume samples (e.g., purified protein analytes^62^ for structural or early-stage molecular analysis). Acoustic droplet ejection is a complex and dynamic process, but three main factors generally determine the dynamic range of the droplet volume at a single ejection event: (1) excitation signal properties, (2) ejector designs, and (3) liquid properties. These three factors play important roles during the ejection process, including the amount of kinetic energy delivered to the drop, the acoustic focal point size, and the resistance of releasing a droplet from liquid surfaces. The upper bound of throughput for on-demand acoustic droplet ejection is empirical and is preeminently limited by the restoration time of the liquid surface after ejection. A detailed discussion is provided in the Methods section and Supplementary Note S3.

Precision cell/particle dispensing is the basis for high-resolution single-cell experiments. When a virtual fluidic channel and a multicolor single-cell detection unit are coupled to the ejector, single-cell nanodrops can be ejected deterministically upon synchronization between the optical detection and acoustic droplet ejection (Fig. 3h). The injected cells are focused into a thin stream of single cells that flow through the laser detection point *via* the inlet capillary. Then, a single cell of interest is ejected upward following a specific time delay when passing through the acoustic focal point. Notably, a virtual fluidic channel is established within the gap between the inlet and the outlet due to the fine-tuned pressure differentials, allowing the cell stream to strictly maintain its shape when flowing through the gap in an open fluidic space (Supplementary Fig. S3 and Movie S3). The device integration is described in detail in the “Methods” section. The doublet-cell-droplet percentage is typically lower than 1% and can be reduced at lower input cell concentrations. On the basis of this mechanism, single-cell nanodrops can be ejected on demand deterministically. By repetitively depositing different types of cells at the same location, precise number control is allowed in individual nanodrops (Fig. 3i, j and Supplementary Movie S4), enabling the possibility of constructing artificial communities with single-cell resolution. Once the nanodrop array is inverted, the particles will be paired or clustered together because of droplet curvatures. These dispensing capabilities allow PULSE to robustly anchor experiments on single cells.

### Maintaining the native states of biological samples *via* PULSE

We managed to minimize the shocks introduced by PULSE to maximize the potential of maintaining the native states of biological samples. First, the working frequency (i.e., 20.4 MHz) chosen is within the range of prenatal ultrasound imaging. Second, mechanistically, upon an ejection event, the short acoustic pulse (e.g., 1 ms) primarily actuates the fluids surrounding the cell for encapsulation and ejection instead of actuating the cell itself. Third, the mechanical impact is minimized since the ejected nanodrop has a low terminal speed of 0.7 m⋅s^−1^. Fourth, cavitation-induced reactive oxygen species are not detected (Supplementary Note S4), which is a major concern for high-intensity focused ultrasound applications^63^. Finally, to avoid evaporation, the dispensing speed and volume of the nanodrops (e.g., 200 nL) are tuned to prevent osmotic shocks to the cells.

**Fig. 4:**
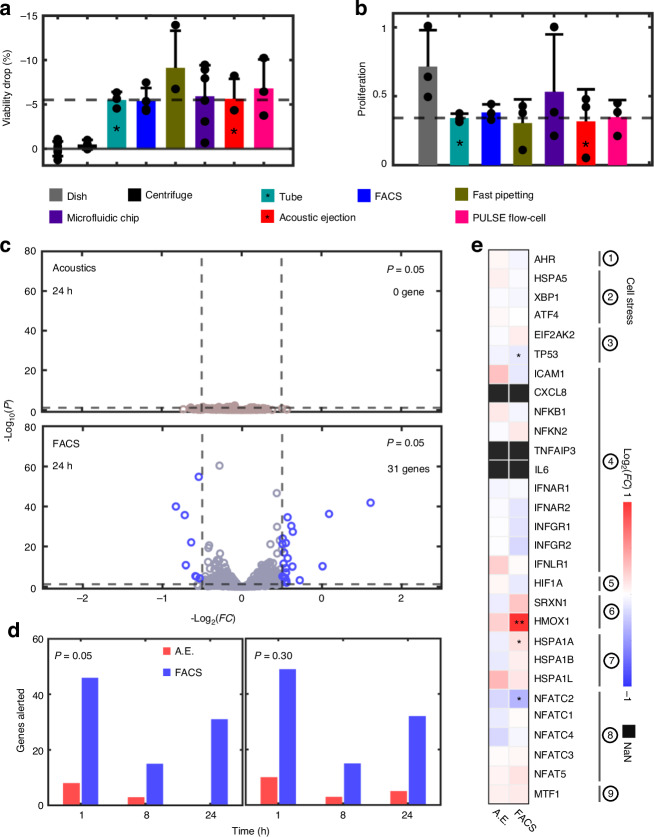
Biocompatibility of PULSE. **a** Comparison of cell viability in samples processed via different methods at 1 h. “*”, bar of interest. Dashed line: threshold anchoring on the “tube” condition. **b** Comparison of the proliferation rates of samples processed by different technologies over a 72-h period. **c** Genes differentially regulated in cells dispensed by PULSE and fluorescence-activated cell sorting (FACS) at 24 h, *P*_adj_ = 0.05. Dashed line: vertical, fold change >1.5; horizontal, *P*_adj_ < 0.05. **d** Time-lapse count of genes differentially regulated by PULSE and FACS. Left, *P*_adj_ = 0.05. Right, *P*_adj_ = 0.30. **e** Comparison of the effects of the cell stress panel on cells processed via PULSE and FACS at 24 h. ①–⑨: panels on gene regulation, endoplasmic reticulum stress, DNA damage, inflammation, hypoxia, oxidative stress, heat shock, osmotic stress, and metal stress, respectively. “*”, *P*_adj_ < 0.05. “**”, *P*_adj_ = 0.001. The genes of interest are identified via RNA sequencing in (**c**–**e**). A.E. acoustic ejection, FC fold change

For calibration, the adherent cell line MCF7 was used to systematically examine the biocompatibility of PULSE. As shown in Figs. 4a and 4b, an array of state-of-the-art cell dispensing methods, such as fluorescence-activated cell sorting (FACS) and microfluidic chips, are compared with PULSE from the perspectives of the cell viability (1 h) and proliferation rate (0–72 h), suggesting that PULSE is as gentle as pipetting the detached cells into an Eppendorf tube. Since FACS is the gold standard for cell processing, we used RNA sequencing to compare PULSE and FACS for differentially regulated genes. The acoustically ejected cells demonstrated no significant differential gene regulation within 24 h postinjection (normalized to cells without any treatment other than detachment; see “Methods”). In contrast, FACS revealed alterations in 31 genes across different panels (Fig. 4c). Different time slices (i.e., 1, 8, and 24 h) were examined (Fig. 4d) to distinguish short-term and long-term shocks, which indicated that PULSE is significantly safer than FACS even under a loose statistical standard (*P* < 0.30). A closer look at the cell stress panel revealed that the gold-standard method resulted in numerous significant gene expression changes in pathways such as DNA damage (TP53), oxidative stress (HMOX1), heat shock (HSPA1A), and osmotic stress (NFATC2). In contrast, cells processed with PULSE showed no notable stress response. These findings highlight the minimal interference and high biocompatibility of PULSE, making it suitable for applications that involve delicate biological samples, such as enzymes, mammalian cells, and model organisms.

### Configuring biofabrication *via* PULSE

**Fig. 5:**
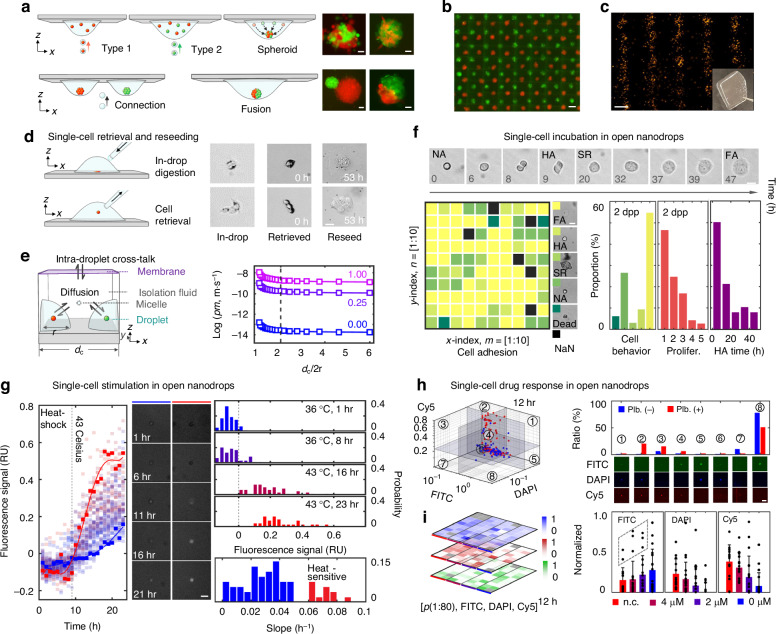
Biofabrication and precision gating *via* PULSE. **a** Fabrication of hybrid spheroids from HEK293T and NIH/3T3 cells in inverted nanodrop-pixels via simple cascade logic. Scale bar: 20 µm. **b** Two-dimensional deposition of bioinks in chess format. Scale bar: 600 µm. **c** A hydrogel slice containing a printed pattern. Scale bar: 600 µm. **d**–**i** Precision gating *via* PULSE. **d** In-drop digestion, retrieval, and reseeding of single cells. Scale bar: 20 µm. **e** Incubation chamber design for maintaining humidity and minimizing diffusion-induced cross-contamination. Right, calibrated permeability curve of isolation fluorinert (0%, 0.25%, and 1% surfactant) with respect to the relative distance of the nanodrops. **f** Long-term tracking of single-cell behaviors from the nanodrop-pixel array. FA, full-adherence. HA, half-adherence. SR, spheroid. NA, nonadherence. **g** Heat shock and apoptosis kinetics monitoring of single cells in the nanodrop-pixel array. Red, heat-sensitive cells. Blue, heat-resistant cells. Scale bar: 20 µm. **h** Single-cell drug sensitivity screening of palbociclib. FITC, green, cell-membrane permeability. DAPI, blue, cell-membrane reversion. Cy5, red, DNA binding. Plb., palbociclib. Plb. (−), 0 μM. Plb. ( + ), 16 μM. Scale bar: 30 µm. **i** Single-cell responses to the palbociclib gradient. Dashed box, potentially drug-resistant single cells. n.c., negative control; 16 μM. Scale bar: 20 µm

Owing to the spatiotemporal programmability of PULSE, bioinks can be deposited following simple printing logic to fit different biofabrication scenarios with high precision and reconfigurability. For example, by sequentially depositing two types of cells (i.e., embryonic kidney and fibroblast stem cells) in an exact location, hybrid spheroids can be formed in inverted droplets in a scalable manner. Fabricating fused spheroids is also feasible via staged deposition (Fig. 5a). In addition to spheroids, PULSE allows the 1D and 2D patterning of cells on the basis of the deposition of different bioinks (Fig. 5b) and the transfer of patterns to hydrogel slices (Fig. 5c). Moreover, PULSE presents the opportunity to construct artificial cellular communities through precise control of the number of particles within nanodrops. These capabilities suggest that PULSE has significant potential in biofabrication-related fields, including tissue engineering, embryogenesis, and synthetic biology.

### Precision gating with single-cell resolution *via* PULSE

As a pilot test, single-cell nanodrops were deposited on a biocompatible substrate for on-chip incubation and multicolor time-lapse imaging for an extended period (>50 h). This test revealed population-level heterogeneity and rare events of cell behaviors. We also demonstrated that the cell of interest can be retrieved from the open substrate via a 1 µL pipette for downstream operations. Note that to retrieve adherent cells from the nanodrops, an extra in-drop digestion step is needed, after which the cells can be reseeded and regrown in bulk medium (Fig. 5d).

Nanodrops are advantageous for increasing throughput and precision and confining cell factors over bulk-fluid experiments. In contrast to droplet microfluidics, PULSE prints nanodrops onto open substrates instead of confining the droplets to microchannels, resulting in four key advantages: (1) allowing cells to adhere, (2) easy cell retrieval, (3) free from surfactant-induced cross-contamination, and (4) relatively large volume for cell incubation (72 h *vs*. 8 h). The first two are important for many biomedical assays, particularly those demanding long-term incubation of adherent cells while maintaining cell health. However, incubating single cells in open nanodrops without affecting cell integrity and health is challenging. For PULSE, an array of factors, including temperature, humidity, oxygen, and carbon dioxide levels, are fine-tuned for the incubation device since the nanodrops are highly sensitive to environmental fluctuations.

Among these factors, evaporation is the critical bottleneck for single-cell incubation and can easily interfere with experiments through osmotic shocks. A humidity chamber (Fig. 5e, left) was carefully designed to host the substrate and nanodrops for long-term cultivation to avoid such shocks. Specifically, the nanodrop array is immersed in an oxygen-permeable, water vapor-permeable, and water-immiscible inert fluorinert fluid and then capped with a gas-permeable PDMS membrane with the medium on the top, which serves as a humidity barrier and a water vapor pressure buffer, respectively. Another interfering factor is the crosstalk between nanodrops, which prevents droplet-based assays from handling small-molecule drugs or further scaling up. In our incubation experiments, unlike droplet microfluidics technologies^64–66^, surfactants are not needed for phase stabilization since the nanodrops are not in contact with each other. Thus, the undesired micelles, diffusion, and cross-talk^67^ are minimized (Fig. 5e, right; Supplementary Note S5), enabling a high level of orthogonality between nanodrops.

On the basis of these features, PULSE can reveal single-cell heterogeneity and rare events with minimal shocks from the hardware. As a result, owing to the fine-tuning of the microenvironment, ejected single-cell MCF7 cells can adhere to and proliferate normally within the open nanodrops during incubation for 48–72 h (Fig. 5f and Supplementary Movie S5). For the cell stimulation scenario (i.e., thermodynamic treatment), as shown in Fig. 5g and Supplementary Movie S6, heat shock-induced single-cell apoptosis was continuously tracked to establish kinetic curves with statistical power, revealing heat-resistant cancer cell subgroups. As a demonstration of precision drug screening, single breast cancer cells and the FDA-approved CDK 4/6 inhibitor palbociclib were printed for incubation and monitoring. Figure 5h shows a 3D scatter plot of the single-cell responses of DNA-binding (Cy5), cell-membrane permeability (FITC), and reversion (DAPI) upon treatment, where each octant represents a type of cell behavior potentially linked with different mechanisms. Reagents can also be preloaded onto the substrate and cryo-dried for subsequent cell screening or mass storage. As shown in Fig. 5i, a substrate with a preloaded palbociclib gradient was tested, revealing the drug-resistant subgroups and the dose effect in a single experiment.

### Deterministic array barcoding with droplet-addressable primers *via* PULSE

**Fig. 6:**
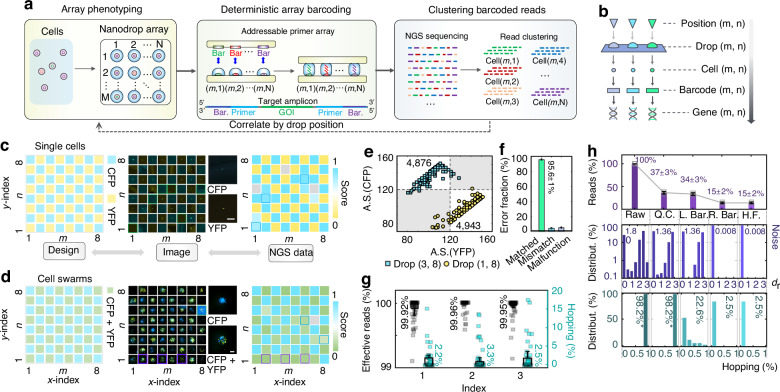
Deterministic array barcoding of plasmid genes with addressable primers *via* PULSE. **a** Schematic workflow. Nanodrop-pixels are transferred to a primer array with known barcode sequences to corresponding positions to enable deterministic tagging and clustering of reads. The structure of the derived amplicon is shown in the lower middle panel. “GOI,” genes of interest. “Bar,” barcode. **b** Schematic concept of our deterministic array barcoding strategy. **c** Direct correlation between single-cell images and genetic contents. Inset: example images of single *E*. *coli* cells containing the CFP or YFP gene. **d** Direct correlation between the images of yeast swarms and their genetic contents. Inset: example images of yeast swarms containing CFP or CFP-YFP genes. “NGS data,” next-generation sequencing data, gated by thresholds of alignment scores >120. Green box, imaging defects. Blue box, amplification defects. The purple box indicates the printing defects. “Score,” a normalized metric for profiling genetic content on the basis of the ratio between the cluster numbers of the YFP and CFP genes. **e** Alignments of reads from specific nanodrops hosting a CFP cell and a YFP cell. “A.S.”, alignment score using the Smith‒Waterman algorithm. **f** Error analysis of the deterministic array barcoding. The data are derived via the threshold for generating Fig. 6c. “Matched,” the NGS data matches the cell images among the functional nanodrops (i.e., threshold score: 120). “Mismatch,” the next-generation sequencing data mismatch with the cell images among functional nanodrops. “Malfunction,” malfunctioned nanodrops. **g** Characterization of barcode hopping (blue) and the proportions of high-fidelity reads among the barcode-filtered reads (black) for each barcode. Index, experimental index. **h** Distributions of the fraction of effective reads (upper panel), noise (middle panel), and barcode hopping (lower panel) with respect to the bioinformatic procedures. “Q.C.”, quality control (i.e., Phred quality score >20, 151 bp, 0 base mismatch). “1^st^ Bar.”, reads filtered using the 1st barcode. “2^nd^ Bar.”, reads filtered using the 1^st^ and 2^nd^ barcodes. “H.F.”, high-fidelity reads that are within a genetic distance of 10 with respect to the reference point CFP_0_ (111, 151) or YFP_0_ (151, 111) in the 2D alignment score space. *d*_r_, the minimal relative genetic distance between a read and the CFP or YFP gene, which is normalized by the genetic distance between the CFP and YFP. “Distribut.”, percentage of normalized distribution. “Hopping,” barcode hopping percentage among high-fidelity reads for each barcode. “*”, estimated value. Scale bars: 20 µm

A limitation of the current barcoding methods^68^ is the inability to correlate the sequencing data to specific cells deterministically, mainly owing to the inherent uncontrollability and stochasticity in barcoding experiments during multiple steps, including barcode pooling (e.g., split-and-pool), cell-primer encapsulation, and high-amplification noise in the emulsion. To accurately identify the corresponding cell for each barcode, we developed a technique called “deterministic array barcoding” on the basis of the PULSE methodology. Essentially, barcoding substrates containing multiplexed primer dots were batch-fabricated via contact printing, and each contact position had predried primers with known, unique sequences. As shown in Fig. 6a, the cells were compartmentalized by PULSE and imaged in the 2D droplet array; thus, the acquired phenotypes were registered to the positions (“addresses”) of the nanodrops. Prior to barcoding, amplification cocktails were deposited onto the barcoding substrate *via* PULSE to resuspend the dry barcoding primers drop-by-drop. The nanodrop array was then merged with the barcoding substrate through manual alignment and immersed in oil for subsequent open-chamber thermocycling. After amplification, the products were collected from the open substrate *via* centrifugation for next-generation sequencing (“Methods”). The generated sequencing reads were subsequently clustered by the preacknowledged barcode sequences and correlated to the nanodrops or cells at specific positions. In this way, the gap between phenotypes and genotypes is directly bridged with single-cell resolution (Fig. 6b) via the physical addresses of the nanodrops in the array. Notably, since the barcode sequences and the liquid handling procedures are transparent, more stringent quality control is allowed for the droplets and sequencing reads (e.g., disregarding the reads of broken droplets after visually checking the amplified droplet array).

As a proof-of-concept calibration, single *E*. *coli* cells (Fig. 6c) and yeast swarms (Fig. 6d) containing two types of fluorescence genes (i.e., CFP or YFP genes) were dispensed in a chess format and then imaged, barcoded, and sequenced following the aforementioned pipeline. The spatial distribution of the sequenced genetic contents in both cases matched well with those of the phenotypes and the executed printing design. Importantly, the selected CFP and YFP genes were highly homogeneous (differing only in a few domains); however, they could be effectively distinguished by next-generation sequencing, which was confirmed by the fact that the clustered amplicons derived from these two genes were close in alignment score space (Fig. 6e). For the single *E. coli* scenario, 4.7% of the nanodrops malfunctioned due to experimental defects, and over 95.6 ± 1% of the functional nanodrops presented the expected genetic content (Fig. 6f). In contrast, the remaining 4.4 ± 1% may be due to plasmid loss or amplification failure (“mismatch”). In our bioinformatic analysis, the barcodes on both ends of the amplicons were employed to filter out the “good reads” (i.e., <1% single-base mismatches). After this filtration step, we observed that over 99.9% of the filtered reads were actually “high-fidelity reads” (i.e., genetic distance to CFP_0_ or YFP_0_ < 10) (Fig. 6g). Barcode hopping was validated to be 2.7 ± 0.6% (Fig. 6g); however, the source of the hopping was unclear. As a reference, ~2% barcode/index hopping is expected during standard next-generation sequencing library preparation. To calibrate the uncertainties in the ejection and imaging steps (particularly for small cells such as *E*. *coli*), yeast swarms expressing YFP and CFP proteins were printed in a similar chess format to validate the deterministic array barcoding, which resulted 98.4% functional nanodrops and an accuracy of 96.8%. In addition, the instability of DNA polymerase in nanodrop barcoding was reaffirmed (Supplementary Note S6). Figure 6h shows the characterization of the stepwise distributions of the effective read fractions, relative genetic distances, and hopping per barcode in the single-cell sequencing data-processing pipeline. After filtering using the same barcode sequences on both ends of the amplicons, the variations in genetic distances (i.e., noise) and the accompanying hopping were reduced by 170- and 9-fold, respectively, compared with filtration using only one barcode on one end of the amplicon.

## Discussion

Modern life sciences and biomedical research necessitate tools that are efficient, automated, and capable of high-throughput analysis. These characteristics are essential for deciphering the complex inner workings of living cells. We present PULSE, a compact lab automation solution for quantitative life science research that standardizes large-scale experiments into microscale test matrices via acoustically ejected nanodrops. Unlike traditional lab automation solutions, PULSE focuses on single-cell data for standardization, achieving unparalleled resolution and theoretical significance. In our work, single cells and reagents are programmatically deposited into an array of individually configurable microreactors with high speed, precision, and level of integration. This strategy allows PULSE to seamlessly thread several key levels of biological experiments, from experimental design to phenotype and genotype acquisition, in a deterministic manner. Three major functionalities, namely, biofabrication, precision gating, and deterministic array barcoding, are demonstrated with high precision and controllability *via* PULSE. Compared with existing lab automation methods, PULSE stands out for its ability to achieve high-resolution standardized data production by making single cells the central focus of experiments. Specifically, such standardized single-cell data allow maximal resolution while bypassing the key uncertainties in bulk-cell analyses, including (1) the population mask, (2) unexpected cooperative behaviors, (3) loose statistical approximations, and (4) ambiguities in sample composition and protocol interpretations.

Compared with other mechanisms, including pneumatic-pressured nozzles, piezoelectric inkjets, thermal-bubble inkjets, electrohydrodynamic jetting, and microvalve-based printing, acoustic droplet ejection has advantages such as being nozzle-free (clogging-free), contact-free (multiplexable), label-free, highly biocompatible, and high-throughput. The downsides are that acoustic-based methods typically require a radio-frequency power supply and extra dead volumes to allow waves to focus above the transducer, and it is challenging to eject highly viscous liquids (e.g., honey, glycerol). In addition to the advantages of acoustic droplet ejection, PULSE has additional advantages, such as being miniaturized, scalable, easy to make, offering a higher-frequency range (for smaller droplets), having a large dynamic volume range (200 fL to 10 μL), and being able to dispense relatively viscous samples (e.g., 80% v/v glycerol, 66 cP at 25 °C).

PULSE is highly adaptable and can be integrated with a diverse array of analytical tools, including high-resolution optical imaging, time-of-flight mass spectrometry, Raman spectroscopy, off-substrate bulk cultivation, and other multi-omic technologies, to achieve comprehensive analyses of single cells. Moreover, PULSE can work with most existing substrates for different purposes, such as microplates, cell culture dishes, electron-beam-transparent substrates (e.g., Si_3_N_4_), or Raman-transparent substrates (e.g., CaF_2_).

PULSE is a highly biocompatible laboratory automation solution. It has been meticulously refined to minimize self-interference and maximize the preservation of the native states of biological samples. The PULSE platform allows adherent cells to grow in open nanodrops for up to 72 h while maintaining normal physiological conditions—a feat challenging for current technologies. Moreover, interdroplet contamination, or crosstalk, is significantly reduced in our platform compared with other emulsion-based approaches, allowing high orthogonality between microreactors.

In future studies, to increase the barcoding throughput of PULSE, the barcoding substrate needs to be scaled up (e.g., 10,000 dots per chip for single-cell deposition and amplification) with help from oligo/microarray manufacturers to achieve a similar level of throughput as established technologies. Additionally, the droplet stability of amplification needs to be optimized to reduce the number of byproducts resulting from dysfunctional enzymatic activities (DNA polymerization). Moreover, although PULSE has been proven to be biocompatible for robust cell lines, further characterization needs to be conducted for fragile cell types such as stem cells. To eliminate the restriction on liquid height, in the future, we will employ coupled echo signals and coupled reagent reservoirs to acquire the liquid height in real time and then dynamically align the acoustic focal point to the liquid surface by moving the ejector vertically on a motorized stage. In this way, we could dynamically compensate for the loss of liquid height during dispensing.

Scientific tools are deeply intertwined with science itself. Prominent achievements enabled by scientific tools include the discovery of penicillin with the aid of a microscope and a petri dish and the creation of artificial life by the use of DNA synthesizers and sequencers. New developments in technology have iteratively expanded the horizons of biology. In this context, PULSE was developed as an efficient automation solution for single-cell analysis. PULSE can facilitate quantitative precision biology in the context of embryogenesis, tissue engineering, immunology, evolutionary dynamics, self-driving laboratories, and synthetic biology.

## Methods

### Device fabrication

(1) Fabrication of the interdigital transducers (IDTs). IDTs with frequencies ranging from 10 to 180 MHz are fabricated via straightforward one-layer lithography followed by metal deposition. Three-inch, 0.5 mm thick, double-sided polished *X*-cut LiNbO_3_ wafers (PWLN-131122, Precision Micro-Optics, USA) are spin-coated with positive photoresists (SPR3012, MicroChem, USA) at speeds of 3000–6000 rpm, exposed, and developed following the manufacturer’s protocol. In particular, the orientation of the photolithography mask is matched with the crystal orientation of the wafer correctly according to the crystal cut information of the wafer’s manufacturer (-*X* surface in this case). Subsequently, 5 nm Cr and 200 nm Au are deposited on the patterned wafer *via* an E-Beam evaporator (Solution, CHA Industries, USA), and the wafers underwent a standard lift-off process to reveal the metallic IDT patterns (e.g., 3-mm-diameter metallic ring-combs with a 1.1-mm-diameter hollow region for the 20.4 MHz acoustic cell ejectors). The IDTs are then treated with plasma and immersed in 5% (3-aminopropyl)-triethoxysilane (APTES, Milliopore-Sigma, USA) water solution at 90 °C for 60 min. The treated wafers are then exposed to plasma again and coated with an insulation layer (2–8 μm exposed SU8 photoresist, Microchem, USA) with the APTES adhesion layer to accommodate conductive liquids. These IDTs are subjected to electrical and optical quality control procedures and attached to wires via silver epoxy. The detailed design principle of the ejector IDTs is discussed in Supplementary Note S2. The detailed theoretical discussions are presented in Demirci’s and Hadimioglu’s pioneering works^69,70^ on related ejector setups. (2) Fabrication of the flow cell of a single-cell dispenser. The functions of the flow cell include 3D cell focusing, introducing cells to the ejection point, single-cell detection, and removing undesired cells that flow through. Two pieces of polydimethylsiloxane (PDMS) with complimentary structures are bonded together to form the 3D sheath flow nozzle, optical fiber slots, and coaxial inlet and outlet channels (Fig. 3h) for inserting glass capillaries (OD 200 μm, ID 100 μm, 8510, VITROCOM, USA). Note that a virtual fluidic channel is created at the gap between the inlet and outlet capillaries, allowing cells to be confined in free fluid for acoustic ejections. The detailed designs are shown in Supplementary Fig. S3. (3) Assembly of the PULSE cartridge. The cartridge used in this work consists of four acoustic ejectors: three are for reagent/cell-swarm dispensing, and the remaining ejector is for single-cell dispensing with laser detection. 3D-printed structures are aligned and bonded to the LiNbO_3_ wafer to form four independent reservoirs and confine the fluid meniscuses and water waves. The geometrical centers of the virtual fluidic channel and the ejector IDT are aligned in the plane to achieve robust cell encapsulation, forming a complete cartridge.

### System integration

(1) Optical instruments. For single-cell detection, a 488 nm laser (Pavilion Integration Corp., USA) serves as an excitation source and is coupled to the cartridge through optical fibers (M43L and M15L, ThorLabs, USA). The scattered and fluorescence signals are collected and directed to photomultiplier tubes (PMTs, H10721-20, Hamamatsu Inc., Japan) for multichannel detection. For postejection imaging, PULSE interfaces with an automated inverted microscope (Ti-Eclipse, Nikon, Japan) with a motorized stage and humidity chamber for large-scale, multicolor imaging. (2) Electronic instruments. The radio-frequency power supply includes a function generator (AFG3102C, Tektronix, USA) and an amplifier (25A250A, Amplifier Research, USA). This function generator is for gating pulse signals whose frequency is lower than 50 MHz. An oscilloscope (MSOX 2024 A, Keysight, USA) is employed to gather cell signals from PMTs and trigger the functional generator. A control box (USB-6001, National Instruments, USA) is used to tune the gain values of the PMTs. (3) Fluidic instruments. Pneumatic pumps (370 air pump, Baenrcy, USA) inject cells into and withdraw waste from the flow cell in customized configurations through silicone tubes. Cells are loaded into the PULSE system in Falcon tubes in a manner similar to that of a conventional cytometer. (4) Motorized stage. The motorized stage is modified from a low-cost 3D printer (Anet A8, AENT3D, China) with customized controlling units (Arduino UNO, CNC shielding board, and A4988 stepper driver). This stage allows on-demand 3D translation with a minimal *x‒y* distance of 13 μm and 29 steps⋅s^−1^ for 900 μm steps with tolerable shaking at high moving speeds. (5) Software integration. All the components are integrated into the PULSE system via the customized software myBase built upon the application designer in MATLAB 2020a (MathWorks Inc., USA). myBase interfaces with users and synchronizes over 100 printing parameters and associated hardware for an experiment.

### System operation

(1) Acoustic droplet ejection. First, 432 μL of fluid is added to the reservoir (dimensions: 14 mm × 21 mm) via a pipette. Then, 20.4 MHz, 80–140 V_pp_ sinusoidal pulses (0.5–10 ms) are applied to the ejector IDT with a repetition rate of up to 200 Hz. The distance between the air‒water interface is preferably less than 9 mm to maintain ejection directivity. The initial volume of added fluid can be reduced to as low as 0.3 μL when smaller reservoirs and high-frequency IDTs are used (Supplementary Movie S7). Echo-based fluid height sensing is feasible (Supplementary Fig. S4). (2) Single-cell ejection. Cells at a concentration of 1–5 × 10^6^ particles⋅mL^−1^ in 50 mL Falcon tubes are injected into the cartridge at 5.5 psi with a coaxial sheath flow under a pressure of 5.0–5.5 psi. The acoustic pulse duration and delay are 1.2–2 ms and 150–250 μs, respectively. The detection of single cells is based on the peak widths of fluorescence signals instead of edge detection since slower/faster moving particles are not ejected accurately. (3) Test matrix. Multiple parameters of a test matrix, including the row number, column number, coordinates, and repetition number, are input into the myBase software in table format. Then, the myBase software translates this test matrix to execution codes for the PULSE system.

### Cell culture and preparation

(1) Cell samples. MCF7 cells (MCF7, ATCC, USA) were cultured in Dulbecco’s modified Eagle’s medium (DMEM) supplemented with 10% fetal bovine serum and antibiotics. *E. coli* cells (Top10, Invitrogen, USA) were cultivated in LB broth supplemented with antibiotics to maintain the plasmids. Metabolically deficient *S. cerevisiae* (Δ*ura*-, ATCC, USA) were transformed with plasmids and cultivated in the corresponding synthesized selection media for protein expression. The high-expression yeast cells were enriched by a fluorescence-activated cell sorter and reseeded to an agar plate prior to cell harvesting. The algal cells were obtained from pond water. Five-day-old *C. elegans* worms were obtained from Prof. Joel Meyer’s laboratory at Duke University. The spheroids were derived from MCF7 cells via the hanging drop method. Fixed *D. rerio* embryos were obtained from the Zebrafish Core Facility. (2) On-chip incubation of nanodrops. Substrates of various materials, including PDMS, glass, and PMMA, were coated with an adherence layer by immersing them into 1 mg⋅mL^−1^ type-I collagen from rat tails (A1048301, Thermo Fisher, USA). Immediately after printing, the substrate was immersed in FC-40 fluorinert oil (3 M, USA) presaturated with water vapor and 5% CO_2_. Specifically, for single mammalian cells, a conditioned medium (e.g., new-to-old ratio of 1:1) is preferred to increase cell growth. The immersed substrate was then placed in a customized incubation chamber and an on-microscope container (INU, TOKAI HIT, Japan) to maintain proper humidity and gas concentrations for long-term cultivation.

### Biocompatibility validation

MCF7 cells were used to characterize the biocompatibility of PULSE. For Fig. 4a, b, “Dish” refers to cells in a dish without any treatment; “Centrifuge” refers to cells detached and centrifuged at 500 rpm for 5 min; “Tube” refers to cells detached and stored in a tube in the medium; “FACS” refers to all cells detached and sorted by Astrio Sorter (Beckman Coulter, USA); “Fast pipetting” refers to cells detached and fast-pipetted 20 times using a 1000 μL pipette; “Microfluidic chip” refers to cells detached and subjected to flow through of the microfluidic focusing unit using the same fluidic parameters in the “System operation” section; “Acoustic ejection” refers to cells detached and acoustically ejected using the optimal conditions in Fig. 3d; and “PULSE flow-cell” refers to cells detached and subjected to the whole single-cell ejection process in PULSE. All the samples under different conditions were synchronized before analysis. (1) Viability. Double-staining (calcium green AM and ethidium homodimer, Thermo Fisher Scientific, USA) and flow cytometry were used to characterize the degree of cell membrane integrity after treatment. (2) Proliferation. The treated cells were reseeded on dishes and monitored for 96 h. The confluency of the dishes was measured via large-scale imaging and image processing via ImageJ and MATLAB. The relative proliferation rate is the average daily confluency difference between 96 h and 24 h. (3) RNA-Seq. The RNA contents of triplicate samples under the “acoustic ejection,” “FACS,” and ejector reservoir (without acoustics) conditions were extracted (RNeasy Plus Mini Kit, Qiagen, USA), sequenced (NovaSeq 6000 S-Prime 50 bp PE, Illumina, USA), and analyzed (Duke Bioinformatics Core). Three posttreatment time points (1, 8, and 24 h) were evaluated to reveal short- and long-term effects on the cells. The run statistics are summarized in Supplementary Table S1.

### Nanodrop crosstalk validation

A 50 μL 100 μM Rhodamine 6 G (Millipore-Sigma, USA) drop and a 50 µL water drop (r = 2.5 mm) were placed on the bottom of a reservoir with a certain separation distance (*d*_c_ = 25.4 mm). Different isolation fluids, including air, mineral oil (molecular grade, BioUltra, Millipore, USA), silicone oil (5 cSt, Millipore, USA), FC-70 (3 M, USA), and FC-40 (3 M, USA) with 0%, 0.25%, and 1% 008-fluoropolymer surfactants (RAN technologies, USA), were used to characterize the diffusion in the isolation fluids and the water drops after different incubation periods (1.5 h, 4.3 h, and 23 h) (Supplementary Fig. S5). After incubation, the fluorescence signals from 40 μL of isolation fluid and 40 μL of the corresponding water drop were measured via a plate reader (525 nm/560 nm, Synergy H1, BioTek, USA). The diffused Rhodamine 6G concentrations were then calculated from a previously established calibration curve and fitted into the diffusion model to obtain the permeability.

### Ejection performance characterization

(1) Volume characterization. Ejected nanodroplets were deposited on a hydrophobic substrate. The substrate was subsequently immersed in mineral oil (molecular grade, BioUltra, Millipore Sigma, USA) with 5% Span 80 (Millipore Sigma, USA) to obtain spherical shapes for image recognition-based measurements. Large-scale microscopy images were obtained to measure the nanodrop diameters and calculate the droplet volumes. For calibration, larger nanodrops were loaded into capillaries with known diameters, which allowed the droplet volume to be derived by measuring the length of the wetted region in the capillary. “*Volt*._0_” in the manuscript is defined as 110 Vpp, “*Vol*._0_” as 42 nL. (2) Directivity characterization. Nanodrops were printed in 1D format, and the position of each nanodrop was derived via a similar image-processing method. The deviations of the positions were then calculated and divided by the standard step length of 1.2 mm to obtain the relative deviation of the directivities. For the manual pipetting shown in Fig. 3g, the standard step length was set to 9 mm, which is the spacing of a 96-well plate. (3) Image stitching correction. Owing to the stitching imperfections of large-scale microscopy images, the related errors must be corrected. To this end, calibration stencils with a periodicity identical to that of the nanodrop array were processed similarly, and the inherent errors were obtained and then compensated. (4) Hydrophone measurement. A whole wafer with the ejector to be measured was fixed at the bottom of a water reservoir, and a hydrophone (HGL0085, Onda Corporation, USA) was mounted on a programmable XYZ stage synchronized with a function generator (AFG3102C, Tektronix, USA) and an oscilloscope (MSOX 2024 A, Keysight, USA). Sections in the *x*–*y* plane (*z* = 2.5 mm), *x*–*z* plane (*θ* = 135°), and *y*–*z* plane (*θ* = 225°) were raster-scanned, forming a 3D intersection view within a 2 mm cubic box with a 1 mm vertical distance to the geometric center of the transducer. Closer distances to the wafer make disentangling acoustic pulse signals from electromagnetic interferences in the time domain challenging. The cross-sections of the acoustic pressure color maps are shown in Fig. 3a with their rotational relationships to the ejector and the employed piezoelectric material.

### Biofabrication *via* PULSE

HEK293T embryonic kidney cells and NIH/3T3 embryonic fibroblasts were stained (Cell Tracker Green/Orange, Invitrogen, USA) and printed into hanging drops. Owing to the curvature of the hanging drops, the cell swarms spontaneously clustered together and formed spheroids. The construction of a large-scale chess pattern was based on the printing of MCF7 cells with two types of cell tracker dyes (periodicity: 850 μm).

### Precision gating *via* PULSE

(1) Heat shock. Single MCF7 cells in a nanodrop array were continuously monitored for 23 h for the expression of an apoptosis marker (Annexin V CF350, Biotium, USA). Notably, at 9 h, the incubation temperature was increased to 43 °C. Heat-sensitive subpopulations were derived from the average fluorescence slope from 9 h to 16 h. (2) Drug gradient screening. Palbociclib (Millipore Sigma, USA) was diluted to 4 μM, printed onto a substrate with gradients of repetition numbers, and then lyophilized for storage. Single MCF7 cells were then deposited on the same substrate in an aligned manner. The apoptosis and cell death monitoring dyes were carefully chosen to achieve long-term observation and avoid spectral cross-talk. The fluorescence channels for Fig. 5h were as follows: FITC, calcium AM green (Invitrogen, USA); DAPI, Annexin V CF350 (Biotium, USA); and Cy5, DRAQ7 (Thermo Fisher Scientific, USA).

### Deterministic array barcoding *via* PULSE

(1) Primer design and synthesis. As a proof-of-concept, barcoding primers were designed to amplify target genes. Six nucleotide barcodes were inserted into the forward and backward primers, with each pair of primers having unique barcodes. These barcoding primers were directly synthesized following standard primer specifications for amplicons (IDT technologies, USA). The priming sites for secondary amplification were attached to the 5’-ends of both the forward and the backward primers. (2) Barcoding chip fabrication. An 8-by-8 PDMS microwell array (with a height of 250 µm) was fabricated through standard soft lithography, hydrophobically treated, and loaded with drops of known primers to form the mother chip. The mother chip was then aligned with another daughter chip, and specific amounts of liquids were transferred to each microwell of the daughter chip through contact printing. In this way, the daughter chips could be fabricated in batches. The fabricated barcoded chips were then dried for long-term storage at 0 °C. (3) Constructing plasmids with target genes. The plasmid containing the target YFP gene (PSF-TEFI-TPI1-YFP-URA3) was purchased from Millipore Sigma. We constructed a similar plasmid with the target CFP gene due to delays obtaining the CFP counterpart from the manufacturer. The CFP gene was synthesized (GenScript, USA) and inserted into the same plasmid backbone via Golden Gate assembly (NEB). Notably, yeast and *E. coli* can host these plasmids, but the reporter genes are only promotable in yeast. Before being printed for barcoding, the yeast cells were digested with zymolyase (2 µL of enzyme in 100 µL of resuspension buffer, Zymo Research, USA) at 37 °C for 45 min. Then, the digested cells were resuspended in a 9.5% osmotic sucrose solution. *E*. *coli* cells were harvested from liquid culture with antibiotics; cells with CFP plasmids were stained with CellBrite® Fix 488 (Biotium, USA), and cells with YFP plasmids were stained with both CellBrite® Fix 488 and CellTracker™ Orange CMRA dyes (Thermo Fisher, USA). Pseudocolors were added to the cell images. (4) Barcoding procedure. Cells with two types of genetic contents (i.e., plasmids) were printed in chess format on a substrate and imaged under a microscope. Then, 400 nL of 0.89 × Taq-polymerase master mix (M0496, NEB, USA) was added to each spot of the cell-loaded substrate. Note that the concentration of the master mix was slightly lower than 1× (i.e., 0.89×) to compensate for evaporation during handling and amplification. Finally, this substrate was aligned with and merged with the barcoding substrate to transfer the droplets upon capillary effects. For the single *E*. *coli* experiments, cell and master-mix droplets were directly deposited on the barcoding substrate for imaging and amplification. Each well in the loaded barcoding chip contained all the components for amplification. For thermocycling, the loaded barcoded chip was immersed in a thin aluminum box filled with mineral oil (BioUltra, Millipore Sigma) with humidity control and coupled to a conventional thermal cycler (S1000, Bio-Rad, USA) via a thermal coupling gel. Notably, the temperature curve was calibrated to compensate for the extra materials on the thermal cycler. A detailed discussion is provided in Supplementary Note S7.

### Sequencing and bioinformatics for deterministic array barcoding

The amplified nanodrops on the PULSE chip were collected by centrifugation at 3000 rpm in 50 mL Falcon tubes. The collected products were delivered to the sequencing core of the Duke Center for Genomic and Computational Biology for quality control, purification, library construction (KAPA HyperPrep, Roche, USA), and next-generation sequencing (MiSeq v2 Micro 150 bp PE, Illumina, USA). Notably, during library construction, preamplification (typically ~6 rounds) after adapter ligation was used to efficiently enrich the target bands in the gel electrophoresis images but this resulted in noticeable barcoding hopping due to the remaining unused barcoding primers in the library aliquot. The generated fastq files were processed via the MATLAB Bioinformatics toolbox for trimming, quality control, barcode splitting, alignment, and analysis. The detailed run statistics are shown in Supplementary Table S2, and the list of DNA oligonucleotides is summarized in Supplementary Table S3.

## Supplementary information

Supplementary Movie S1

Supplementary Movie S2

Supplementary Movie S3

Supplementary Movie S4

Supplementary Movie S5

Supplementary Movie S6

Supplementary Movie S7

Supplemental Material File #1
