# Supplementary material for: Automating life science labs at the single-cell level through precise ultrasonic liquid sample ejection: PULSE
Source: Microsyst Nanoeng. 2024 Nov 20;10:172. doi: 10.1038/s41378-024-00798-y (PMC11579414; doi:10.1038/s41378-024-00798-y)
Supplement: Supplementary file 8 — Supplemental Material File #1 [file 41378_2024_798_MOESM8_ESM.docx]

Supplementary Information

**Automating life science labs at the single-cell level through precise ultrasonic liquid sample ejection: PULSE**

Peiran Zhang^a^, Zhenhua Tian^b^, Ke Jin^a^, Kaichun Yang^a^, Wesley Collyer^a^, Joseph Rufo^a^, Neil Upreti^a^, Xianjun Dong^c^, Luke P. Lee^d,e,f,g*^, and Tony Jun Huang^a,*^

^a^ Department of Mechanical Engineering and Materials Science, Duke University, NC 27708, USA;

^b^ Department of Mechanical Engineering, Virginia Polytechnic Institute and State University, VA 24061, USA;

^c^ Genomics and Bioinformatics Hub, Department of Neurology, Brigham and Women’s Hospital, Harvard Medical School, Boston, MA, USA;

^d^ Department of Medicine, Brigham and Women’s Hospital, Harvard Medical School, Boston, MA, USA;

^e^ Department of Bioengineering, Department of Electrical Engineering and Computer Science, University of California at Berkeley, Berkeley, CA, USA;

^f^ Institute of Quantum Biophysics, Department of Biophysics, Sungkyunkwan University, Suwon, Korea.

^g^ Department of Chemistry & Nanoscience, Ewha Womans University, Seoul, Korea

^*^ To whom correspondence should be addressed. Email: lplee@bwh.harvard.edu, tony.huang@duke.edu

**Supplementary Notes.**

**Supplementary Note S1.** Customized myBase software.

myBase software is developed to synchronize hardware based on the user’s experimental design input. myBase and its associated graphical user interface are compiled using MATLAB (MathWorks, USA). The software is divided into several modules: bottom-layer drivers, hardware communication and monitoring, precision positioning, acoustic pulse control, single-cell signal gating, test matrix translation and visualization, debugging panel, and parameter/experiment log registration. Reagents and single cells are programmatically deposited with the spatiotemporal synchronization of sub-modules to achieve arbitrary experimental design. The detailed composition and organization of myBase software are shown in **Supplementary Figure S1**.

**Supplementary Note S2.** Designing speed-fitting transducers for acoustic droplet ejection.

The schematic principle of wave focusing is shown in **Supplementary Figure S2**. The speed-fitting transducers are designed based on the following constraints:

(1) The wavelengths along different angular directions must match the polar speed distribution of SAWs of the *X*-cut LiNbO_3_ crystal (­-*X* surface in this case).

(2) The direction of IDT electrode stems needs to match with the polar amplitude distribution of the *X*-cut LiNbO_3_ crystal to avoid losing efficiency.

(3) The alternativeness of the metallic fingers needs to match the antisymmetric nature of the used piezoelectric material [1].

(4) The focal point needs to match water thickness, and the leaked waves in all directions should be in-phase at the focal point by tuning the 2D distribution of the IDT fingers.

(5) The wavelength of the transducer limits the minimal volume of droplets to be ejected. Based on experience, the measured diameter of the focal point is around 1.5 times the acoustic wavelength in fluid. For example, 8 - 10 μm diameter water droplets can be ejected using 180 MHz pulse signals in PULSE.

**Supplementary Note S3.** Optimizations done on the liquid dispensing *via* acoustic ejector.

**I. The acoustic ejectors are optimized in the following aspects to enhance robustness.**

**(1) Liquid confinement.** The shape of the reservoir is fine-tuned to achieve optimal fluid meniscus and damp water waves for continuous dispensing.

**(2) Workhorse piezoelectric material.** One major consideration for selecting LiNbO_3_ is to generate higher frequency surface acoustic wave (SAW, specifically in Rayleigh wave mode, *e.g.*, 10 – 180 MHz range) components along the substrate using interdigital transducers (IDTs). With higher frequency waves, the transducer can generate a narrower focal point and smaller droplets that are appropriate for a single-cell microreactor scenario. Although the piezoelectric constant or electromechanical coupling coefficient of LiNbO_3_ is low, these SAWs majorly confine its energy along the substrate surface, and then effectively leak into fluid to form acoustic focal point annularly. In addition to all the physical considerations, the choice of piezoelectric material should also be within the empirical contexts of device fabrication, device robustness, and device durability. For example, we can fabricate ~100 ejectors on a 3-inch polished wafer using a straightforward one-layer photolithography and metal deposition, and the fabricated IDTs stay mechanically robust and electrochemically inert during experiments. During device assembly, the optical transparency of LiNbO_3_ has the added benefit of easier device alignment. The specific cut of the piezoelectric material employed (*X*-cut LiNbO_3_) is selected based on both theoretical prediction and experimental validation among different promising substrates (*e.g.*, *Y*36, *Y*, *Z*-cut LiNbO_3_). Therefore, our acoustic ejectors can work at relatively low voltage (80 – 140 V_pp_ for 20.4 MHz signal) compared with other LiNbO_3_ substrates. Higher input voltage may cause problems, including electromagnetic interferences, power supply instabilities, substrate breakdown, and potential radiation hazards.

**(3) Sensitivity to fluid thickness.** According to our acoustic pressure data (**Fig. 3a**) and the droplet volume data (**Fig. 3f**), the acoustic ejectors are not very sensitive to the fluid thickness around the focal point. Also, as shown in **Supplementary Fig. S4**, echo-based fluid level sensing is possible without changing the ejector design.

**(4) Biocompatibility.** The workhorse frequency employed by PULSE is carefully chosen to avoid detrimental effects on bio-analytes. Further, the chosen droplet volume should resist evaporation within the time frame of printing and chip transferring.

The acoustic ejection process is biocompatible due to the following four factors:

1. **Low Cavitation Level:** Lower frequency ultrasound is known to generate cavitation and related reactive oxygen species (ROS), which are effective in inducing cell stress, aging, and death. To confirm that our higher frequency solution does not induce these ill effects, we conducted a pilot test using Abcam’s Cellular ROS Assay Kit (Supplementary Note S4) on ejected cells. The results indicate that no cell-sensible ROS are generated during acoustic droplet ejection, implying lower levels of cavitation.
2. **Minimal Mechanical Stresses:** The following three factors minimize the chances of mechanical stresses during acoustic ejection:
   1. The employed acoustic wavelength for cell ejection (73 μm at 20.4 MHz) is much smaller than the cell diameter.
   2. The acoustic radiation force is primarily exerted on the surrounding fluid of the cell.
   3. The acoustic contrasts of cells and the fluid are similar.
3. **Negligible Heating:** The acoustic pulses have short durations of around 1 ms, and the focal point is 1.4 mm away from the acoustic transducer. Therefore, heating is not a considerable concern at this stage.
4. **No Steady Acoustic Streaming:** Steady acoustic streaming will not form within the 1 ms acoustic pulse duration. Since cells have similar density and acoustic contrast to the surrounding fluid, the relative acceleration to the surrounding fluid pocket to be ejected is minimal. As a rough estimate, the ejected cells will gain a vertical speed of around 0.7 m/s within 1 ms, leading to an average acceleration of 700 m/s² during the excitation window. This acceleration is equivalent to 70×*g* gravitational force for 1 ms in a centrifuge, which is far below the widely used centrifugation condition of 500×*g* for 5 minutes, known to be safe for cells in biological experiments.

Mechanical index (MI) is a good indicator for measuring the bioeffect of acoustic beams. Due to the challenges of directly measuring the acoustic pressure at high voltage excitation signals (80-140 Vpp) due to electromagnetic interference with a hydrophone, we measured the acoustic pressure at an excitation voltage of 10 Vpp (maximally 140 KPa at the focal point). This allows a proportional estimation of 1.12–1.96 MPa for the cell ejection scenario. The derived mechanical index (MI = peak negative pressure (MPa) / frequency^(1/2) (MHz)) should be around <0.43 for the cell ejection scenario, which is lower than the FDA’s maximum threshold of 1.9 for ultrasonic imaging [2].

**II. Other interesting features.**

**(1) Coupled ejection.** Droplets can be dispensed using detachable reservoirs with acoustically transparent bottoms.

**(2) Atomization.** Fluid atomization is achieved at high excitation amplitudes and high repetition rates of excitation signals, resulting in a highly confined beam of fluid droplets upon atomization.

**(3) Minimal dead volumes.** For precious samples like proteins for high-resolution imaging, acquiring several microliters of analytes may require months of effort. Our dispensing technology allows the dead volume to be as small as 0.3 μL, granting minimal consumption in each experiment. Also, the lowest volume of ejection droplets we have achieved is 0.2 pL at a speed of 40,000 drop⋅sec^-1^, which can potentially reduce the sample waste on blotting and allow high-throughput applications for small-scale samples.

**III. Major factors influencing the dynamic range and the throughput of acoustic droplet ejection.**

Three primary factors affect the dynamic range of the droplet volume at a single ejection event.

1. **Excitation signals**: Generally, the higher the frequency, the smaller focal point of the acoustic beam that an ejector can generate, and then the lower the volume of the smallest droplet an ejector could eject. Conversely, higher amplitudes and longer pulses duration will lead to larger droplet volume.
2. **Ejector properties:** The focal distance and the width of the acoustic focal point of an ejector is determined by the ejector dimension, shape, and the angle of leaky waves. Generally, the smaller the focal point, the smaller the droplet; and vice versa. To achieve a narrow acoustic focal point, the overall dimension ejector needs to be smaller while the hollow part of the ring-comb transducer needs to be larger. Also, the thickness of water layer over the ejector needs to be aligned with the acoustic focal point vertically to eject smaller droplets.
3. **Liquid properties:** Generally, lower surface tension and lower viscosity will reduce the lower limit of the dynamic volume range (liquid surface releases droplets more easily), and the higher surface tension and higher viscosity will increase the upper limit of the dynamic volume range (*i.e.*, droplets tend not to break apart by a single ejection event).

Regarding throughput, since we are pursuing on-demand droplet ejection (instead of continuous droplet dispensing), to ensure ejection quality (*e.g.*, volumetric consistency and directivity), the upper bound of throughput of droplet ejection is hard limited by the restoration time of the liquid surface post ejection. For example, for the ejector we employed for PULSE, since the acoustic focal point is large (around 200 microns, leading to 40 nL droplet volume), the restoration time is relatively long (~4 ms), resulting in maximally 250 droplet/s throughput for on-demand droplet ejection. In extreme cases (*e.g.*, in picoliter-ejectors), since the acoustic focal point is extremely small, the liquid surface restoration time is much shorter, allowing for a much higher throughput (up to 40,000 drop/s).

**Supplementary Note S4.** Detection of ultrasound-induced reactive oxygen species (ROS).

It is well-known that high-intensity ultrasound can induce cavitation and associated ROS in fluid, leading to significant oxidative shocks on the cells to be dispensed. Cellular ROS Assay Kit (abcam, USA), MCF7 cells, and H_2_O_2_ standard solutions are employed to characterize the shock induced by ROS. Very minimal changes in ROS-induced responses are observed when comparing with the no-acoustic control group, indicating the biocompatibility of our acoustic ejectors for sensitive cells or fragile reagents.

**Supplementary Note S5.** Cross-talk between droplets.

The cross-talk between emulsion droplets is only mentioned in a few publications, but it is an important barrier for droplet-based applications involving small molecules [3]. According to the literature on droplet microfluidics and digital microfluidics, such cross-talk is due to the high-permeability of reagents in the isolation fluids such as silicone oil and fluorinert, and it is considered a major roadblock for achieving reaction independency. There are two major pathways of reagent permeability: (1) diffusion and (2) micelles upon surfactant-phase interactions. As shown in **Supplementary Figure S5**, among different isolation fluids, fluorinert FC-40 without surfactant has high resistance (< 5 × 10^-4^ %) to cross-contamination of fluorescence dye Rhodamine 6G while holding high oxygen/CO_2_ permeability. Note that Rhodamine 6G can be considered as the worst-case scenario for fluorinert according to the milestone paper on “minimal emulsions.”

**Supplementary Note S6.** Instability of amplification in nanodrops.

DNA amplification in an open nanodrop array is inherently challenging due to the high sensitivity of nanodrops to environmental fluctuations, including evaporation, enzyme polarization at boundaries, surface adsorption, and inhomogeneous heat transfer. This leads to high error rates and false priming compared with amplification in bulk reactions using commercialized PCR tubes, which has been observed in our NGS sequencing data. Note that similar instabilities have been discussed in the literature on droplet barcoding. For the single-cell amplification experiment in **Fig. 6d**, 37% of read clusters passed through the quality control (PHRED score > 20, length 151 bp) and the barcode filtering (double-end barcodes instead of single-end) with zero mismatches, which is comparable to that of current barcoding methods. After amplification, besides the 151 bp target products, by-products near the length range of 90 bp are also generated. These unexpected byproducts and unused primers (~ 40 bp) can be removed easily during the purification process before library construction. As a control, single-cell amplification in nanodrops in a PCR tube with mineral oil is more stable than in an open nanodrop array, and the by-products and unused primers are less. Due to the existence of by-products and the associated competition, the primer concentration is critical for efficiently amplifying target genes from a single-cell and, therefore, should be optimized prior to large-scale experiments.

**Supplementary Note S7.** Deterministic array barcoding.

(1) Amplification device. The amplification device is optimized based on the following aspects.

(i) The substrate with nanodrop array is immersed in mineral oil inside a customized aluminum box.

(ii) This aluminum box has a very thin wall (~ 1 mm) for efficient heat transfer during thermocycling and has water reservoirs for the in-oil humidity control.

(iii) The substrate is coated with amphiphobic materials (hPDMS and Novec 1720) to avoid surface adsorption.

(iv) The temperature curve is calibrated to ensure fast thermal ramping and temperature accuracy near the nanodrop-substrate.

(2) Selection of target genes. The target genes YFP and CFP are variants with high sequence similarity; therefore, they are good proof-of-concept demonstrations for combining NGS with PULSE. Due to this reason, the primer pairs for amplifying the two types of target genes share the same priming sequences.

**Supplementary Tables.**

**Supplementary Table S1.** RNA sequencing run statistics.

| **Sample condition** | **Platform** | **Time point** | **Direction** | **Pass filter yield (bp)** | **Number of pass filter clusters** | **Q30%** | **Average quality score** |
| --- | --- | --- | --- | --- | --- | --- | --- |
| Cell in reservoir | NovaSeq 6000 S-Prime 50bp PE, Illumina | 1 hr | R1 | 1,431,079,278 | 14,030,189 | 94.33 | 36.07 |
|  |  |  | R2 | 1,420,413,138 | 13,925,619 | 94.30 | 36.06 |
|  |  |  | R1 | 1,637,928,648 | 16,058,124 | 94.41 | 36.08 |
|  |  |  | R2 | 1,627,455,900 | 15,955,450 | 94.39 | 36.07 |
|  |  |  | R1 | 1,533,080,502 | 15,030,201 | 94.38 | 36.07 |
|  |  |  | R2 | 1,522,540,740 | 14,926,870 | 94.33 | 36.06 |
|  |  | 8 hr | R1 | 1,815,963,630 | 17,803,565 | 94.25 | 36.05 |
|  |  |  | R2 | 1,742,083,296 | 17,079,248 | 94.35 | 36.06 |
|  |  |  | R1 | 1,494,030,312 | 14,647,356 | 93.82 | 35.98 |
|  |  |  | R2 | 1,602,600,234 | 15,711,767 | 94.26 | 36.04 |
|  |  |  | R1 | 1,871,436,636 | 18,347,418 | 94.48 | 36.08 |
|  |  |  | R2 | 1,503,566,496 | 14,740,848 | 94.56 | 36.09 |
|  |  | 24 hr | R1 | 2,002,067,526 | 19,628,113 | 94.54 | 36.10 |
|  |  |  | R2 | 1,987,047,720 | 19,480,860 | 94.52 | 36.09 |
|  |  |  | R1 | 1,704,992,424 | 16,715,612 | 94.10 | 36.02 |
|  |  |  | R2 | 1,691,861,658 | 16,586,879 | 94.07 | 36.01 |
|  |  |  | R1 | 1,688,643,864 | 16,555,332 | 94.47 | 36.09 |
|  |  |  | R2 | 1,673,717,184 | 16,408,992 | 94.47 | 36.08 |
| Acoustic ejection | NovaSeq 6000 S-Prime 50bp PE, Illumina | 1 hr | R1 | 1,752,450,168 | 17,180,884 | 94.36 | 36.06 |
|  |  |  | R2 | 1,806,016,080 | 17,706,040 | 94.22 | 36.04 |
|  |  |  | R1 | 1,612,339,296 | 15,807,248 | 94.29 | 36.05 |
|  |  |  | R2 | 1,482,717,288 | 14,536,444 | 93.78 | 35.97 |
|  |  |  | R1 | 1,513,533,732 | 14,838,566 | 94.59 | 36.10 |
|  |  |  | R2 | 1,861,840,782 | 18,253,341 | 94.44 | 36.07 |
|  |  | 8 hr | R1 | 1,773,860,988 | 17,390,794 | 94.55 | 36.10 |
|  |  |  | R2 | 1,765,384,176 | 17,307,688 | 94.52 | 36.09 |
|  |  |  | R1 | 1,578,699,492 | 15,477,446 | 94.12 | 36.03 |
|  |  |  | R2 | 1,569,270,918 | 15,385,009 | 94.11 | 36.02 |
|  |  |  | R1 | 1,948,099,938 | 19,099,019 | 94.24 | 36.05 |
|  |  |  | R2 | 1,934,365,434 | 18,964,367 | 94.21 | 36.04 |
|  |  | 24 hr | R1 | 1,605,204,192 | 15,737,296 | 94.44 | 36.09 |
|  |  |  | R2 | 1,593,704,508 | 15,624,554 | 94.41 | 36.09 |
|  |  |  | R1 | 1,394,488,206 | 13,671,453 | 94.06 | 36.02 |
|  |  |  | R2 | 1,381,125,084 | 13,540,442 | 94.02 | 36.01 |
|  |  |  | R1 | 1,458,743,412 | 14,301,406 | 93.55 | 35.94 |
|  |  |  | R2 | 1,447,870,824 | 14,194,812 | 93.49 | 35.92 |
| FACS | NovaSeq 6000 S-Prime 50bp PE, Illumina | 1 hr | R1 | 1,677,113,988 | 16,442,294 | 94.38 | 36.07 |
|  |  |  | R2 | 1,663,651,314 | 16,310,307 | 94.35 | 36.07 |
|  |  |  | R1 | 1,873,264,986 | 18,365,343 | 94.12 | 36.03 |
|  |  |  | R2 | 1,854,474,342 | 18,181,121 | 94.09 | 36.02 |
|  |  |  | R1 | 1,792,352,976 | 17,572,088 | 93.80 | 35.97 |
|  |  |  | R2 | 1,781,725,698 | 17,467,899 | 93.76 | 35.96 |
|  |  | 8 hr | R1 | 1,691,292,192 | 16,581,296 | 94.39 | 36.07 |
|  |  |  | R2 | 1,677,843,900 | 16,449,450 | 94.36 | 36.06 |
|  |  |  | R1 | 1,702,016,166 | 16,686,433 | 94.24 | 36.05 |
|  |  |  | R2 | 1,689,455,988 | 16,563,294 | 94.20 | 36.04 |
|  |  |  | R1 | 1,602,571,062 | 15,711,481 | 94.22 | 36.05 |
|  |  |  | R2 | 1,591,280,478 | 15,600,789 | 94.20 | 36.04 |
|  |  | 24 hr | R1 | 1,479,506,736 | 14,504,968 | 94.41 | 36.07 |
|  |  |  | R2 | 1,471,244,940 | 14,423,970 | 94.39 | 36.07 |
|  |  |  | R1 | 1,687,753,710 | 16,546,605 | 94.42 | 36.08 |
|  |  |  | R2 | 1,676,609,700 | 16,437,350 | 94.39 | 36.07 |
|  |  |  | R1 | 1,708,628,520 | 16,751,260 | 94.27 | 36.04 |
|  |  |  | R2 | 1,698,688,212 | 16,653,806 | 94.25 | 36.03 |

**Supplementary Table S2.** DABC DNA sequencing run statistics.

| **Sample** | **DABC primer concentration** | **Platform** | **Pass filter yield (bp)** | **Number of pass filter clusters** | **Q30%** | **Average quality score** |
| --- | --- | --- | --- | --- | --- | --- |
| Single *E*. *coli* cell chess pattern 1 | 80 × dilution to stock solution | MiSeq v2 Micro 150 bp PE, KAPA HyperPrep | 105,057,646 | 347,873 | 84.90 | 34.47 |
| Single *E*. *coli* cell chess pattern 2 | 80 × dilution to stock solution | MiSeq v2 Micro 150 bp PE, KAPA HyperPrep | 120,725,708 | 399,754 | 86.71 | 35.43 |
| Single *E*. *coli* cell chess pattern 3 | 80 × dilution to stock solution | MiSeq v2 Micro 150 bp PE, KAPA HyperPrep | 92,396,296 | 305,948 | 84.81 | 34.89 |
| Yeast swarm chess pattern 1 | 40 × dilution to stock solution | MiSeq v2 Micro 150 bp PE, KAPA HyperPrep | 119,594,718 | 396,009 | 89.29 | 34.50 |

**Supplementary Table S3.** List of DNA oligonucleotides and constructs.

| PSF-TEFI-TPI1-YFP-URA3 | Data available at Millipore Sigma* |
| --- | --- |
| Synthetized CFP insert | See the sequence in table note** |
| Golden Gate assembly insert primer 1 | 5’-CACATACATAAACTAAAAATGGCC-3’ |
| Golden Gate assembly insert primer 2 | 5’-GATAATATTTTTATATAATTATATTAACCTTACTG-3’ |
| Golden Gate assembly vector primer 1 | 5’-CAGTAAGGTTAATATAATTATATAAAAATATTATC-3’ |
| Golden Gate assembly vector primer 2 | 5’- GGCCATTTTTAGTTTATGTATGTG-3’ |
| Barcoding forward primer*** | 5’-GACTGGTTCCAATTGNNNNNNAAGAGCGTACCATCACCT-3’ |
| Barcoding backward primer*** | 5’-GCAAATGGCATTCTGNNNNNNAAACCCTGGCCGTTCAGT-3’ |
| 2^nd^ amplification primer 1 (optional) | 5’-GACTGGTTCCAATTG-3’ |
| 2^nd^ amplification primer 2 (optional) | 5’-GCAAATGGCATTCTG-3’ |

* Target YFP gene sequence:

5’-ACGGCACTGACTGAAGGCGCAAAACTGTTCGAGAAAGAAATCCCATATATCACTGAGCTG

GAAGGTGACGTTGAAGGTATGAAGTTTATCATCAAGGGTGAAGGCACCGGTGACGCGAGCGTCGGTAAAGTGGATGCTCAGTTCATTTGTACCACGGGCGACGTTCCGGTTCCGTGGAGCACGCTGGTCACCACGCTGACGTATGGTGCTCAGTGCTTTGCCAAGTATCCGCGCCACATTGCGGATTTCTTCAAAAGCTGCATGCCGGAAGGTTACGTCCAAGAGCGCACCATCACCTTTGAGGGTGATGGCGTGTTCAAGACCCGTGCGGAAGTCACCTTTGAAAATGGCAGCGTGTACAACCGTGTAAAACTGAACGGCCAGGGTTTCAAGAAGGACGGCCACGTGCTGGGCAAAAATCTGGAGTTTAACTTTACCCCTCATTGTTTGTACATTTGGGGTGACCAAGCGAATCATGGCCTGAAGAGCGCGTTCAAAATCATGCATGAGATCACCGGCTCCAAAGAGGATTTCATTGTTGCCGATCACACCCAAATGAATACCCCGATTGGTGGTGGTCCGGTGCACGTGCCGGAGTACCACCACATTACGTATCATGTTACCCTGTCTAAAGACGTCACCGATCACCGTGACCATTTGAACATTGTTGAGGTGATCAAGGCAGTTGACCTGGAGACGTACCGC-3’

** Synthetized CFP insert sequence, which is homogenous to the YFP gene:

5’-CACGGCATTGACGGAAGGCACCAAACTGTTTGAAAAGGAAATCCCGTATATCACTGAAC

TGGAGGGCGACGTCAATGGTATGAAGTTTACCATTCATGGTAAAGGTACTGGCGATGCGACCACGGGCACCATTAAAGCGAAATACATCTGTACTACGGGCGACCTGCCGGTCCCGTGGGCAACCCTGGTGAGCACCCTGAGCTACGGTGTTCAGTGTTTCGCCAAGTACCCGAGCCACATCAAGGATTTCTTTAAGAGCGCCATGCCGGAAGGTTATGTTCAAGAGCGTACCATCACCTTTGAAGGCGACGGCGTGTTTAAGACGCGTGCTGAGGTTACCTTTGAAAACGGTTCTGTCTACAATCGTGTCAAACTGAACGGCCAGGGTTTTAAGAAAGACGGTCACATTCTGCGTAAGAACGTTGCATTCCAATGCCCGCCAGATATGGTGTATATTCTGCCTGACACCGTTAACAATGGCATCCGCGTTGAGTTCAACCAGGCGTACGATATTGAAGGTGTGACCGAAAAACTGGTTACCAAATGCAGCCAAATGAATCGTCCGTTGGCGGGCTCCGCGGCAGTGCATATCCCGCGTTATCATCACCTGAGCAAACACACCAAACTGAGCAAAGACCGCGACGAGCGCCGTGATCATATGTGTCTGGTAGAGGTCGTGAAAGCGGTTGATCTGGACACGTATCAGTAAGGTTAATATAATTATATAAAAATATTATC-3’

*** NNNNNN, barcoding hexamer. The sequences include “TTTTAC”, "TTTTAG”, “TTTATT”, “TTTATC”, “TTTATG”, “TTTATA”, “TTTAAT”, “TTTAAC”, “TTATTC”, “TTATTG”, “TTATTA”, “TTATAT”, “TTATAC”, “TTATAG”, “TTAATT”, “TTAATC”, “TTAAAT”, “TTAAAC”, “TTAAAG”, “TTAAAA”, “TATTTC”, “TATTTG”, “TATTTA”, “TATTAT”, “TATTAA”, “TATATT”, “TATAAT”, “TATAAC”, “TATAAG”, “TATAAA”, “TAATTT”, “TAATTC”, “TAATAC”, “TAATAG”, “TAATAA”, “TAAATT”, “TAAATC”, “TAAATG”, “TAAATA”, “TAAAAC”, “ATTTTG”, “ATTTAT”, “ATTTAC”, “ATTTAG”, “ATTTAA”, “ATTATT”, “ATTATC”, “ATTATG”, “ATTAAG”, “ATTAAA”, “ATATTT”, “ATATTC”, “ATATTG”, “ATATTA”, “ATATAC”, “ATATAG”, “ATAATC”, “ATAATG”, “ATAATA”, “ATAAAT”, “ATAAAC”, “ATAAAG”, “AATTTT”, and “AATTTC”.

**Supplementary Figures.**


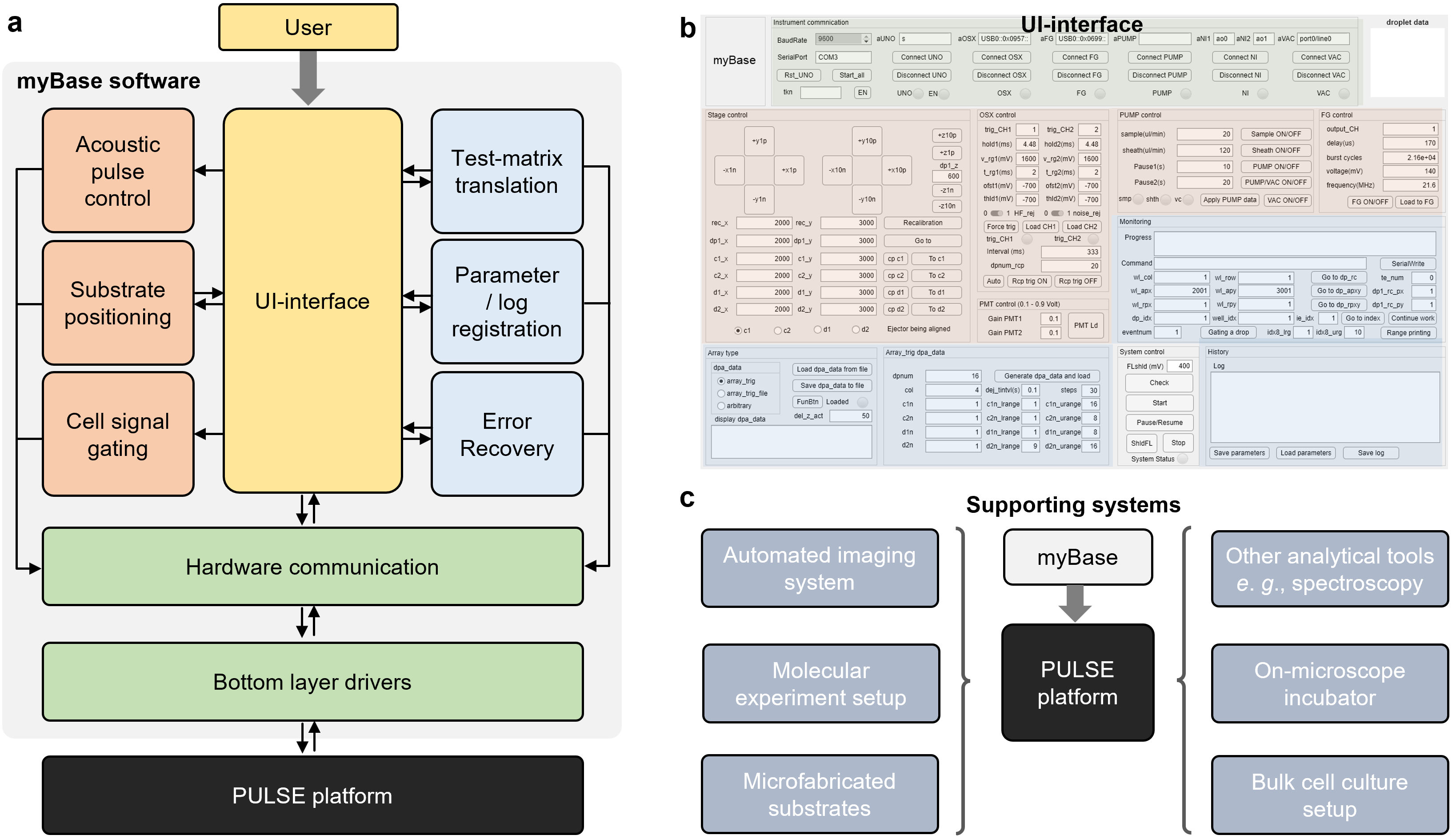


**Supplementary Figure S1.** Illustration of myBase software. (**a**) Modules involved in myBase software and their relationships. (**b**) User interface of myBase software. The color shadings correspond to the modules in **a**. (**c**) The exemplary supporting systems for the PULSE platform to interface upstream sample preparation and downstream analysis using well-established protocols.


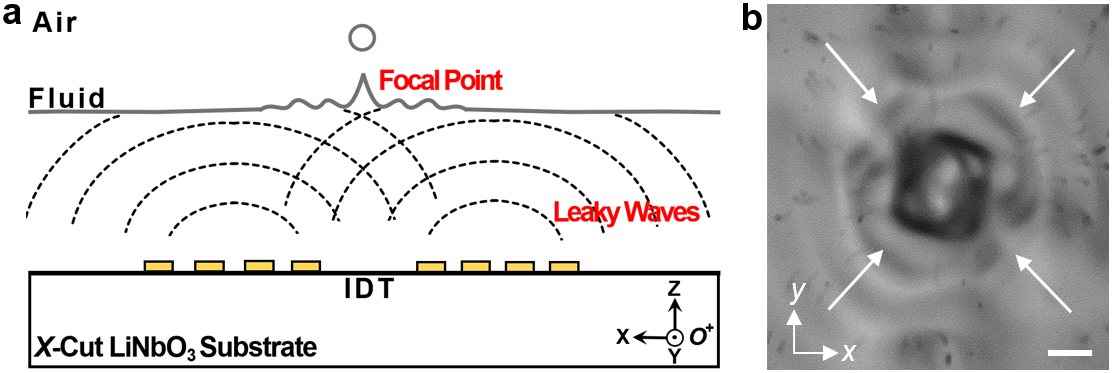


**Supplementary Figure S2.** Schematic principle of acoustic focusing upon leaky wave interferences. (**a**) Cross-sectional side view of the acoustic ejectors immersed in fluid. (**b**) Top view of the surface deformation of a water layer captured by a fast camera. The white arrows indicate the movement directions of polystyrene tracking particles. Scale bar: 80 μm.


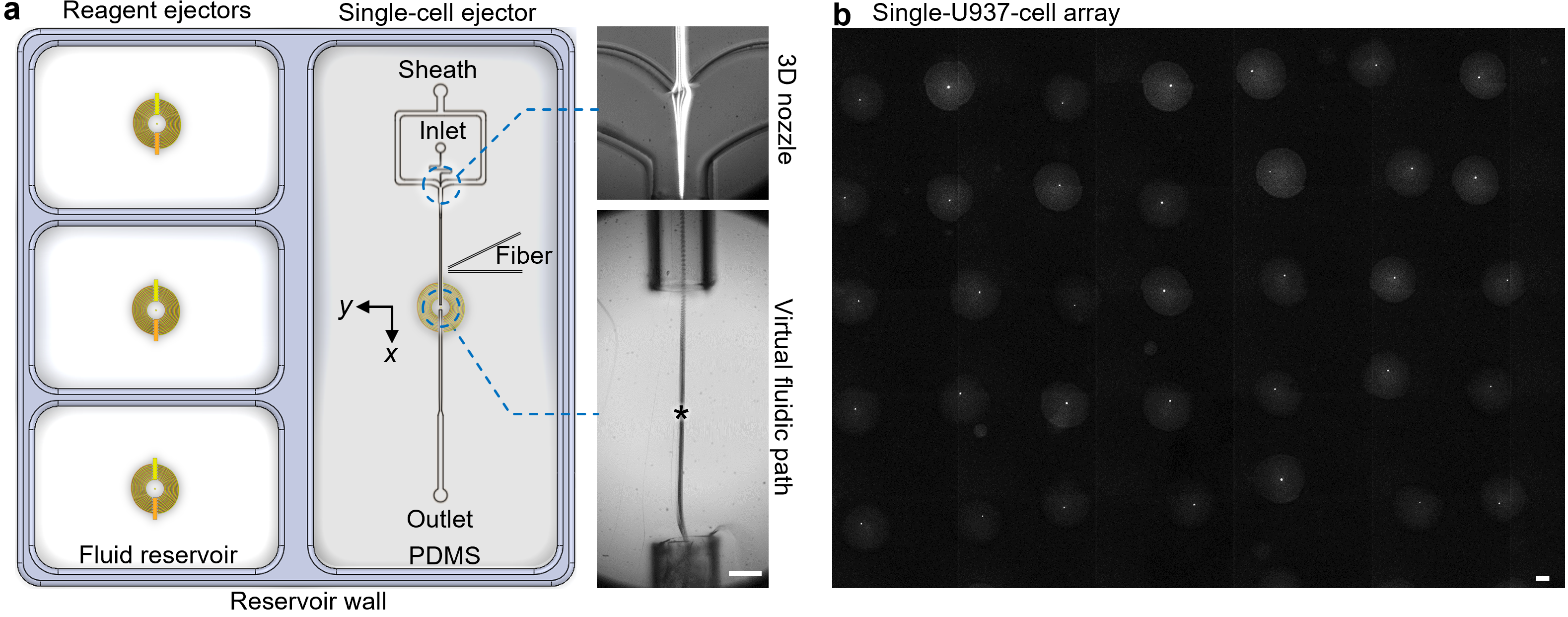


**Supplementary Figure S3.** Design of acoustic-based single-cell ejector in PULSE. (**a**) Schematic top view of PULSE cartridge. Insets: stacked particle-tracking images of 3D sheath nozzle and virtual fluidic path. Asterisk: aligned ejection point. (**b**) Stitched fluorescence image of printed single-U937-cells in DMEM droplet array using single-cell ejector. Scale bars: 100 μm.


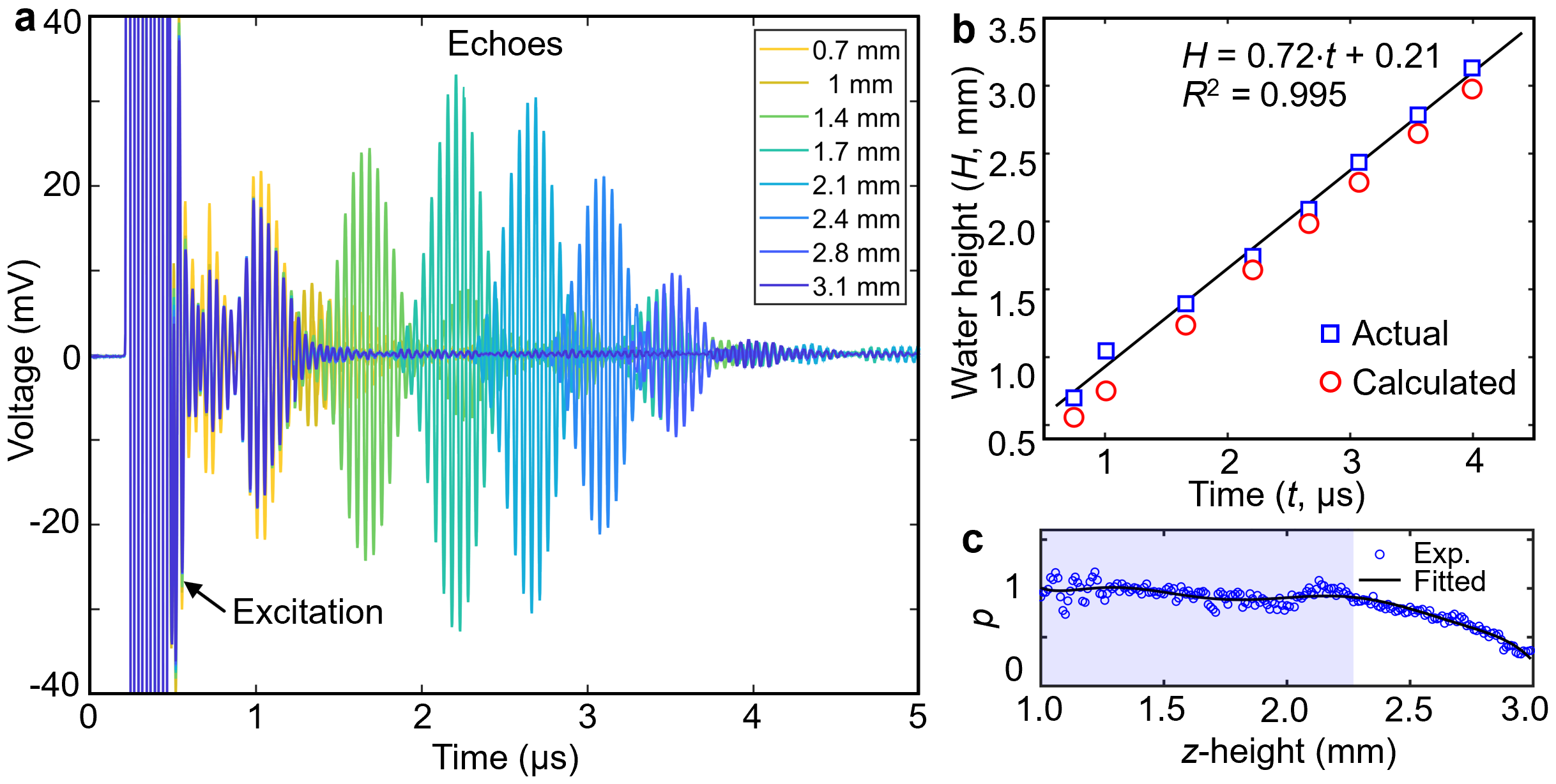


**Supplementary Figure S4.** Echo-based fluid level sensing. (**a**) Example echo signals received upon different water heights. (**b**) The fitted relationship between the merged pulse traveling time (*t*) and the water height (*H*). The traveling time is defined as the difference between the corresponding time-points where the amplitude envelopes of excitation and echo reaching the local maximums. “Actual,” is the water height calculated from the added volume of water. “Calculated,” the water height predicted by the pulse traveling time. The slight mismatch may be due to the water meniscus, sound speed variation, signal envelope deformation, and so on. *c*_water_ = 1,490 m⋅s^-1^. (**c**) Normalized acoustic pressure along the focal axis of the speed-fit transducer (*f* = 20.4 MHz) measured by hydrophone by averaging measurements sampled within an 80 μm by 80 μm area. Blue circles: normalized measured acoustic pressure. Black line: polynomial fit for measured pressure data. The blue shading indicates the range of water height that the axial pressures are insensitive to. Before normalization and averaging by area, the peak acoustic pressure at the focal point is ~140 kPa for a 10 V_pp_ signal set on the functional generator AFG3102C.


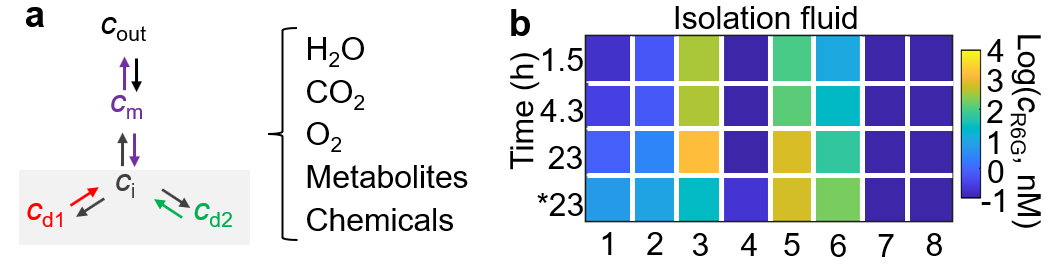


**Supplementary Figure S5.** Cross-talk between two adjacent nanodrops. (**a**) Schematic model of cross-talk. Gray shading: components immersed in isolation fluid. *c*, concentration vectors of different small molecules, including H_2_O, CO_2_, O_2_, metabolites, and added chemicals. “m,” PDMS membrane. “out,” space outside the PDMS membrane. “i,” isolation fluid. “d,” nanodrops. (**b**) Time-lapsed diffusion characterization of different isolation fluids. The detailed experimental setup is enclosed in the **Methods** section.**(*)** The diffused concentrations in the receiving water droplets. “1”, mineral oil (BioUltra, Millipore Sigma, USA). “2”, 5 cSt silicone oil. “3,” mineral oil with 1% Span 80 surfactant. “4”, FC-40. “5”, FC-40 with 1 % fluoropolymer-008 surfactant. “6”, FC-40 with 0.2 % fluoropolymer-008 surfactant. “7”, FC-70. “8”, air. Notably, a high concentration of diffusion in the isolation fluid does not necessarily mean that a high concentration of diffusion in the receiving water droplet. The detailed explanation can be found in supplementary reference 2.

**Supplementary Movies.**

**Supplementary Movie S1.** A time-of-flight simulation video on the side view of wave generation and interference above the transducer. The geometrical center of the transducer is at (0,0). Substrate: LiNbO_3_. (0 – 500 μm thick) Fluid: water (500 – 3000 μm thick). Amplitude: 20 Vpp. IDT period: 200 μm.

**Supplementary Movie S2.** Microscopic view of acoustic actuation on air-water interface at 10,000 drops per second.

**Supplementary Movie S3.** Microscopic view of single particle ejection. A 10 µm particle is focused on the center of the upper capillary, flows through the gap (*i*.*e*., virtual fluidic path), and then is ejected by the 1-ms-duration acoustic waves. The horizontal alignment between the geometric centers of the ejector and the virtual fluidic path does not need to be perfect since the empirical size of the ejection area is around 200 μm. Also, due to the horizontal flow speed component of the fluid-pocket to be ejected, we expect the ejected droplet to have a tilted trajectory. The lateral drifting of the droplet shadow post ejection could be due to optical misalignment, unleveled water surfaces, or unexpected acoustics-fluidics-surface-tension interactions on the capillary, where the last of these could be alleviated by increasing the length of the virtual fluidic path.

**Supplementary Movie S4.** Printing nanodrop array on a motorized stage. (1) Single-particle droplet printing into aligned microwell array. (2) Dual-particle droplet array printing.

**Supplementary Movie S5.** A normal versus a rare cell adhesion/proliferation events of MCF7 in nanodrops. Left: a single-cell adheres and proliferates normally. Right: a single-cell proliferates, forms spheroid first, and then adheres to the substrate. Frame interval: 0.5 hr.

**Supplementary Movie S6.** Time-lapsed heat-shock-induced apoptosis process of MCF7 cells in nanodrops.

**Supplementary Movie S7.** Sub-nanoliter droplet printing with 140 MHz signal. A moving slide is placed over the ejector.

**Supplementary References**

[1] Tian, Z., Wang, Z., Zhang, P., Naquin, T.D., Mai, J., Wu, Y., Yang, S., Gu, Y., Bachman, H., Liang, Y. and Yu, Z., 2020. Generating multifunctional acoustic tweezers in Petri dishes for contactless, precise manipulation of bioparticles. *Sci. Adv.*, **6**, eabb0494.

[2] Şen, T., Tüfekçioğlu, O. and Koza, Y., 2015. Mechanical index. *Anatolian J. Cardiol.*, **15**, 334.

[3] Gruner, P., Riechers, B., Semin, B., Lim, J., Johnston, A., Short, K. and Baret, J.C., 2016. Controlling molecular transport in minimal emulsions. *Nat. Commun.*, **7**, 10392.
